# *Fol*‐milR1, a pathogenicity factor of *Fusarium oxysporum*, confers tomato wilt disease resistance by impairing host immune responses

**DOI:** 10.1111/nph.17436

**Published:** 2021-05-30

**Authors:** Hui‐Min Ji, Hui‐Ying Mao, Si‐Jian Li, Tao Feng, Zhao‐Yang Zhang, Lu Cheng, Shu‐Jie Luo, Katherine A. Borkovich, Shou‐Qiang Ouyang

**Affiliations:** ^1^ College of Horticulture and Plant Protection Yangzhou University Yangzhou JS 225009 China; ^2^ Department of Microbiology and Plant Pathology Institute for Integrative Genome Biology University of California 900 University Avenue Riverside CA 92521 USA; ^3^ Joint International Research Laboratory of Agriculture and Agri‐Product Safety of Ministry of Education of China Yangzhou University Yangzhou JS 225009 China

**Keywords:** *Fusarium oxysporum* f. sp. *lycopersici*, immunity response, plant–pathogen interactions, resistant gene, tomato wilt disease, trans‐kingdom miRNA

## Abstract

- Although it is well known that miRNAs play crucial roles in multiple biological processes, there is currently no evidence indicating that milRNAs from *Fusarium oxysporum* f. sp. *lycopersici* (*Fol*) interfere with tomato resistance during infection.
- Here, using sRNA‐seq, we demonstrate that *Fol‐*milR1, a trans‐kingdom small RNA, is exported into tomato cells after infection.
- The knockout strain *∆Fol‐milR1* displays attenuated pathogenicity to the susceptible tomato cultivar ‘Moneymaker’. On the other hand, *Fol‐*milR1 overexpression strains exhibit enhanced virulence against the resistant cultivar ‘Motelle’. Several tomato mRNAs are predicted targets of *Fol‐*milR1. Among these genes, *Solyc06g007430* (encoding the CBL‐interacting protein kinase, *SlyFRG4*) is regulated at the posttranscriptional level by *Fol‐*milR1. Furthermore, *SlyFRG4* loss‐of‐function alleles created using CRISPR/Cas9 in tomato (‘Motelle’) exhibit enhanced disease susceptibility to *Fol*, further supporting the idea that *SlyFRG4* is essential for tomato wilt disease resistance. Notably, our results using immunoprecipitation with specific antiserum suggest that *Fol‐*milR1 interferes with the host immunity machinery by binding to tomato ARGONAUTE 4a (SlyAGO4a). Furthermore, virus‐induced gene silenced (VIGS) knock‐down *SlyAGO4a* plants exhibit reduced susceptibility to *Fol*.
- Together, our findings support a model in which *Fol‐*milR1 is an sRNA fungal effector that suppresses host immunity by silencing a disease resistance gene, thus providing a novel virulence strategy to achieve infection.

## Abstract 2

See also the Commentary on this article by Thieron *et al.*, **232**: 464–467.

## Introduction

*Fusarium* *oxysporum* is a ubiquitous soil fungus that causes vascular wilt disease in > 100 plant species, including tomato (Pietro *et al*., 2003; Ouyang *et al*., 2014). Tomato wilt is one of the most significant diseases affecting tomato production (Goswami & Kistler, 2004). *Fusarium oxysporum* grows in the vascular bundles in the plant host, from the parasitic phase to the saprophytic phase when conidia are produced, leading to wilt symptoms of the infected plants. Germination of dormant conidia in soil results in adherence and invasion of plant roots by fungal hyphae. The movement of hyphae from the host root cortex to the xylem vessels is critical for disease progression, which is difficult to control and becomes a major threat to plant growth and tomato production (Validov *et al*., 2011b).

Plants are exposed to many external stimuli, including abiotic (e.g. drought, salinity, temperature) and biotic (e.g. microbes, nematodes, insects) stresses (Jones & Dangl, 2006; Atkinson & Urwin, 2012). Recognition of microbe‐associated molecular patterns (MAMPs) by plant pattern‐recognition receptors (PRRs) leads to pattern‐triggered immunity (PTI) in plants. Pathogens have evolved secreted effector proteins to undermine PTI. In return, plants evolve disease resistance (R) proteins, such as nucleotide‐binding leucine‐rich repeat (NB‐LRR)‐type receptor‐like proteins, to directly or indirectly recognize the presence or action of specific effectors, and to activate effector‐triggered immunity (ETI), which is well known as the second layer of the immune response (He *et al*., 2007; Boller & Felix, 2009; Boller & He, 2009).

Small RNAs (sRNAs) are noncoding single‐stranded RNAs that are 20–30 nucleotides in length (Huang *et al*., 2019). Micro RNAs (miRNAs) are a class of sRNAs that originate from the primary miRNA transcripts (pri‐miRNAs) transcribed by RNA polymerase II (Pol II). The single‐stranded pri‐miRNAs are processed into precursor miRNAs (pre‐miRNAs) and exported by RNA‐dependent RNA polymerases (RDR). Pre‐miRNAs are then sliced into sRNA duplexes by Dicer‐like (DCL) proteins. One strand of the sRNA duplex is incorporated into Argonautes (AGOs) to form RNA‐induced silencing complexes (RISCs). Finally, RISC complexes repress the expression of target genes via cleavage of transcripts, inhibition of translation or DNA methylation (Baulcombe, 2004; Baldrich & San Segundo, 2016; Feng *et al*., 2021).

RNA interference (RNAi), triggered by small RNAs (sRNAs) such as small interfering RNAs (siRNAs) and miRNAs, is a well characterized mechanism for modulation and fine‐tuning of plant immunity genes (Ruiz‐Ferrer & Voinnet, 2009; Katiyar‐Agarwal & Jin, 2010; Schwessinger & Ronald, 2012). Plant endogenous siRNAs and miRNAs (e.g. miR393 and miR863 in Arabidopsis, miR482 and miR5300 in tomato) have been shown to regulate genes important for plant defense during the response to pathogens (Navarro *et al*., 2006; Ouyang *et al*., 2014; Niu *et al*., 2016). Many pathogens of plants and animals secrete small molecules (e.g. nutrients, proteins, nucleic acids) during infection (Horbach *et al*., 2011). In pathogens, effector proteins are secreted into their host plants to suppress innate immunity in order to promote successful infection (Stergiopoulos & de Wit, 2009; Kombrink & Thomma, 2013; Asai & Shirasu, 2015). The first reported pathogen‐derived sRNAs functioning as ‘RNA effectors’ were discovered in the fungal pathogen *B. cinerea* (Weiberg *et al*., 2013). *Botrytis cinerea* delivers sRNAs into Arabidopsis and tomato to perturb the host immune signaling pathways. *Bc*‐siRNAs were shown to associate with plant Argonaute 1 (AGO1) protein to suppress host immunity, as well as to target host plant mitogen‐activated protein kinase transcripts during infection (Weiberg *et al*., 2013). The same team later showed that Arabidopsis cells secrete exosome‐like extracellular vesicles to deliver sRNAs into *B. cinerea* cells to silence genes crucial for pathogenicity (Cai *et al*., 2018). These findings demonstrate that plants have adapted trans‐kingdom RNAi as part of their host immune responses during the evolutionary arms race with the pathogen. Argonaute‐4 (AGO4) is another major nuclear RNAi component that, in contrast to AGO1, has been demonstrated to regulate the disease‐resistance response through DNA methylation, leading to increased plant defense (Bhattacharjee *et al*., 2009; Katiyar‐Agarwal & Jin, 2010).

Since these discoveries in *B. cinerea*, sRNAs have been implicated as effectors in other pathogen or parasite–plant interactions. For example, *Pst*‐milR1, a microRNA‐like (milRNA) gene from *Puccinia striiformis* f. sp. *tritici* (*Pst*), represses the host immune response by suppressing expression of a wheat pathogenesis‐related gene (B. Wang *et al*., 2017). Consistent with the action of *Bc*‐siRNAs, *Pst*‐milR1 may also be exported as an RNA effector to impair the host immune defense response (B. Wang *et al*., 2017). Intriguingly, in contrast to the horizontal transfer of sRNA from pathogen to plant, it has recently been shown that the parasitic plant *Cuscuta campestris* delivers specific 22‐nt miRNAs (*Ccm*‐miRNAs) to an Arabidopsis host. Several Arabidopsis mRNAs are targeted, triggering production of endogenous secondary siRNAs (Shahid *et al*., 2018). These studies suggest that both fungi and parasitic plants can use a trans‐kingdom sRNA mobility strategy to impair the host innate immune system to achieve success during infection.

Calcium (Ca^2+^) is a ubiquitous secondary messenger that functions during the response to abiotic/biotic stresses and developmental processes in plants. Calcineurin B‐like proteins (CBLs) act as the major Ca^2+^ sensors by interacting with CBL‐interacting protein kinases (CIPKs) to form a CBL–CIPK signaling network (McAinsh & Pittman, 2009; Dodd *et al*., 2010). CIPK family members regulate reactive oxygen species (ROS) production during ETI and PTI through a vital connection between Ca^2+^ and ROS signaling under biotic stress conditions (Steinhorst & Kudla, 2013; Liu *et al*., 2018; Tang *et al*., 2020).

Here, we investigate roles for the RNA interference (RNAi) machinery in tomato wilt disease defense. Two near‐isogenic tomato cultivars, ‘Moneymaker’ (susceptible, *i‐2*/*i‐2*) and ‘Motelle’ (resistant, *I‐2*/*I‐2*), were used. The *I‐2* gene of tomato confers resistance to the race 2 strain of *Fusarium oxysporum* f. sp. *lycopersici* (*Fol*) (Simons *et al*., 1998). The *I‐2* locus encodes a coiled‐coil (CC) NB‐LRR protein that recognizes the Avr2 effector from *Fol* (Houterman *et al*., 2009). We evaluated the ability of a potential sRNA effector, *Fol‐*milR1, to be transferred from *Fol* to a tomato host plant during infection. We investigated the role of *Fol‐*milR1 in pathogenicity of *Fol*, including how *Fol‐*milR1 regulates the tomato target gene *SlyFRG4* (encoding the CBL‐interacting protein kinase), and impairs host immunity by binding to ARGONAUTE 4a (SlyAGO4a).

## Materials and Methods

### Plant materials, fungal inoculation, measurements of *Fol* biomass and grading of tomato wilt disease

Two previously described near‐isogenic tomato cultivars, susceptible ‘Moneymaker’ (MM, *i2*/*i2*) and resistant ‘Motelle’ (Mot, *I2*/*I2*), were employed in this study (Ji *et al*., 2018a; Ouyang *et al*., 2014). Tomato seedlings were grown in long‐day conditions (16 h : 8 h, light : dark photoperiod, at 25°C, 65% humidity, and with a photon flux density 40 μmol m^−2^ s^−1^). Two‐week‐old seedlings were inoculated for all experiments.

The pathogenic fungal strain is *Fusarium oxysporum* f. sp *lycopersici* (race 2) (*Fol*), strain FGSC 9935. *Fol* was grown on potato dextrose agar medium (PDA) for 7 d at 28°C in constant light. Spore suspensions were prepared by harvesting cultures in Vogel's minimal medium (Vogel, 1956) at a concentration of 10^8^ spores ml^–1^. Tomato seedlings were removed from soil, and roots were inoculated with *Fol* spores for 30 min. Water treatment was used as a mock control. All experiments were conducted using three biological replicates.

To assess the relative levels of *Fol* biomass in tomato leaves, genomic DNA was isolated from tomato leaves using cetyl‐trimethyl‐ammonium bromide CTAB (Huang *et al*., 2000). The rDNA intergenic spacer region (IGS) of *Fol* was amplified from genomic DNA using qPCR (primers listed in Supporting Information Table S1: *IGS1049*/*IGS1050*), and used as a marker to assess relative fungal biomass (Validov *et al*., 2011a).

The severity of tomato fungal wilt disease was empirically categorized into five grades during pathogen invasion: 0, healthy plants (no visible wilting or yellowing symptoms); 1, cotyledon wilted or dropped off; 2, 30–50% of the true leaves wilted or dropped off; 3, 50–80% of the true leaves wilted or dropped off; and 4, all leaves dropped off or death of the entire plant. Disease grades were scored at 14 d post‐inoculation (dpi) with *Fol* or water, using 10 individual plants for each treatment (Fig. S1).

### Construction of RNA‐Seq libraries and analysis

Two‐wk‐old tomato seedlings were infected with either mock (water) or *Fol* for 24 h. Three biological replicates were used, with 20 seedlings for each treatment. The roots were rinsed briefly and then frozen immediately in liquid nitrogen. Total RNA was extracted using the TRIzol reagent (cat. no. 15596026; Life Technologies) according to the manufacturer's recommendations. For each Illumina library, 1 μg total RNA was used, according to the manufacturer’s instructions. The libraries were subsequently sequenced using the Illumina HiSeq™ 2000 (Biomarker Technologies, Rohnert Park, CA, USA).

Approximately 60 Mb of raw reads were obtained from each library and then subjected to quality control (QC). After QC, raw reads were filtered into clean reads (18–30 nt sRNAs). All sequence reads were trimmed to remove the low‐quality sequences. The sequence data were subsequently processed using in‐house software tool seqqc v.2.2. Housekeeping small RNAs, including ribosomal RNAs (rRNAs), transfer RNAs (tRNAs), small nuclear RNAs (snRNAs), and small nucleolar RNAs (snoRNAs) were removed by blasting against GenBank (http://www.ncbi.nih.gov/Genbank) servers. The trimmed reads were then aligned to the *Fusarium oxysporum* reference genome using tophat v.2.0.0 and bowtie v.0.12.5 (Maji *et al*., 2014) with default settings. The expression levels of miRNAs were normalized to the reads per million (rpm) value for each individual library.

### sRNA gel blotting, quantitative real‐time PCR and 5′RLM‐RACE assay

For high molecular weight RNA gel blots, 40 µg of total RNA was separated on 7 M urea/15% denaturing polyacrylamide gels in Tris/Boric Acid/EDTA (1× TBE) and subsequently transferred to a nylon N+ membrane. miRNA‐specific oligonucleotide probes (Table S1) were end‐labeled using (γ‐32P)ATP (cat. no. M0201; New England Biolabs, Ipswich, MA, USA; oligonucleotide probes were labeled according to the manufacturer's recommendations). Blots were stripped and re‐probed using a *U6* RNA oligonucleotide probe to provide a loading control. All blots were imaged using a PhosphorImager (Molecular Dynamics/GE Life Sciences, Pittsburgh, PA, USA) (Ouyang *et al*., 2014).

For the *Fol‐*milR1 stem loop reverse transcriptase‐polymerase chain reaction (RT‐PCR), mature *Fol‐*milR1 was reverse transcribed from 1 μg of total RNA using the TaqMan Small RNA Assay kit (cat. no. 4398987; Life Technologies) according to the manufacturer's recommendations (Table S1, *Fol*‐milR1_RT) (Varkonyi‐Gasic *et al*., 2007). Real‐time quantification of miRNAs was performed as described previously (Chen *et al*., 2005; Feng *et al*., 2009). Diluted cDNA was used as the template for quantitative RT‐PCR (cat. no. 1708880; Bio‐Rad), using 18s rRNA as the internal control (Table S1, *Fol*‐milR1_F/Universal_R, *Sly*_18S‐rRNA‐F/*Sly*_18S‐rRNA‐R). Differential expression of genes was calculated using the 2^−∆∆CT^ method (Livak & Schmittgen, 2001).

The 5′RACE assay was performed using the FirstChoice RLM‐RACE kit (cat. no. AM1700; Thermo Scientific, Waltham, MA, USA). The PCR fragments (Table S1, 5′ RACE‐OUTER) obtained from 5′RACE were inserted into the pMD18‐T vector (cat. no. 6011; Takara, Kusatsu, Japan), and individual clones were selected for DNA sequencing.

### Isolation of total RNA from tomato root protoplasts

Isolation of tomato root protoplasts has been described previously (Ouyang *et al*., 2021). Briefly, the roots of 2‐wk old tomato seedlings infected with *Fol* or water (mock) for 24 h were collected and sliced. Sliced roots were immersed in an enzyme solution (3% cellulase R10 (Yakult Honsha, Tokyo, Japan), 1.5% macerozyme R10 (Yakult Honsha), 1% hemicellulase (Yakult Honsha), 0.4 M mannitol, 20 mM KCl, 20 mM MES, pH 5.7) under vacuum for 30 min. Then, samples were incubated for 20 h at 28°C in the dark with gentle shaking (40 rpm on a platform shaker). The protoplast solutions were filtered using a 40 μm nylon mesh Falcon filter to remove undigested root material. The flow‐through solutions were centrifuged at 376 ***g*** at 4°C for 5 min to pellet the protoplasts. Total RNA was then isolated from protoplasts using the TRIzol reagent according to the manufacturer's recommendations.

### Construction of *Fol‐*milR1 knockout, site‐mutated and overexpression strains

*Fol‐*milR1 knockout (KO)/overexpression (OE) mutants and complemented strains were generated by using the split‐marker approach previously described by our laboratory (Li *et al*., 2019a). Briefly, for *Fol‐*milR1 knockout vector construction, the upstream flanking sequence, downstream flanking sequence of pre‐*Fol‐*milR1 and *HPH* cassette were amplified and purified, followed by transformation into protoplasts of the wild‐type strain (Table S1: primers *Fol*‐milR1 (KO)‐1F/*Fol*‐milR1 (KO)‐2R, *Fol*‐milR1 (KO)‐3F/*Fol*‐milR1 (KO)‐4R). Transformants with the desired genetic changes were identified using site‐specific primer pairs (Table S1: primers *Fol*‐milR1 (KO)‐1F/*Fol*‐milR1 (KO)‐HY/R, *Fol*‐milR1 (KO)‐5F/*Fol*‐milR1 (KO)‐8R). For the construction of *Fol‐*milR1 overexpression and complementation vectors, the entire sequence of pre‐*Fol‐*milR1 was inserted under the control of the RP27 constitutive promoter. The fragment was amplified and transformed with XhoI‐digested pYF11 (which confers geneticin resistance) into *Saccharomyces* *cerevisiae* strain XK1‐25 and the final vector was assembled using the yeast gap repair approach (Table S1: primers *Fol*‐milR1_CE_F1/*Fol*‐milR1_CE_R1) (Li *et al*., 2019b). The overexpression and complementation constructs were then transformed into protoplasts of *Fol* and the *Fol‐*milR1 knockout strain, respectively.

To generate the *Fol‐*milR1 site‐mutated (SM) strain, six random nucleotides were introduced into the *Fol‐*milR1 mature region (indicated in Fig. 2d) (Table S1: primers *Fol*‐milR1 (SM)‐1F/*Fol*‐milR1 (SM)‐1R, *Fol*‐milR1 (SM)‐3F/*Fol*‐milR1 (SM)‐4R). Upstream and downstream fragments were obtained by PCR with overlap in the *Fol‐*milR1 mature region. Both fragments were transformed into yeast strain *XK125* and assembled using yeast gap repair followed by construction of a site‐mutated plasmid using the pYF11 vector. *Fol‐*milR1 site‐mutated strains were generated by transformation of the construct into *Fol* protoplasts, as described for the complementation construct above, and verified by sequencing the mutated region.

### Co‐expression of *Fol* milRNAs and predicted mRNA target genes in *Nicotiana benthamiana* leaves

To verify the target of *Fol*‐milR1 in the tomato genome, we performed *Agrobacterium*‐mediated transient co‐expression experiments in *N. benthamiana*, as described previously (Ouyang *et al*., 2014). Briefly, *Fol*‐milR1 and each of its predicted target genes were inserted into vector GATEPEG100. All constructs were transformed into *Agrobacterium tumefaciens* strain GV3101. Transformed *A. tumefaciens* cultures were grown in liquid Luria–Bertani culture medium with selection followed by co‐injection into *N. benthamiana* leaves. In order to inhibit transgene‐induced gene silencing, vector pBAR‐p19BS, containing the TBSV silencing suppressor p19, was co‐infiltrated (generously provided by Prof. Xiao‐Ming Zhang from the Institute of Zoology, Chinese Academy of Sciences) (Lakatos *et al*., 2004). After 36–48 h, the injected leaves were harvested and used for detecting mRNA and protein levels of the target genes, as well as the cleavage sites.

### Generation of *SlyFRG4* loss‐of‐function alleles

The CRISPR/Cas9 (Clustered regularly interspaced short palindromic repeats/CRISPR‐associated 9) system was used to generate a *SlyFRG4* knockout in the resistant tomato cultivar ‘Motelle’ as described previously (Gao *et al*., 2020). Briefly, two adjacent sgRNA target sites within the open reading frame (ORF) of *SlyFRG4* were selected (Naito *et al*., 2015) for insertion into the one‐step binary vector pTX041 using the Golden Gate assembly method (Deng *et al*., 2018) (Table S1: primers *SlyFRG4*_V_F/*SlyFRG4*_V_R, *SlyFRG4*_41_F/*SlyFRG4*_41_R). The final construct was introduced into ‘Motelle’ plants using *A. tumefaciens*‐mediated transformation. Transformants were selected on medium containing hygromycin B (Du *et al*., 2017). Nontransgenic *SlyFRG4* loss‐of‐function homozygous lines were identified in T1 progeny obtained by self‐pollination. Polymerase chain reaction‐based genotyping was carried out on isolated genomic DNA from the T1 progeny, followed by sequencing of genomic DNA using primers flanking both sgRNA target sites (Table S1: primers *SlyFRG4*_Seq_F1/*SlyFRG4*_Seq_R2). Homozygous plants containing the putative loss‐of‐function alleles were identified and employed for downstream phenotypic analysis.

### Virus‐induced gene silencing constructs and phenotype assessment

Virus‐Induced Gene Silencing (VIGS) was utilized to suppress expression of *SlyAGO* genes in the susceptible cultivar ‘Moneymaker’ using TRV‐based vectors (pTRV1 and pTRV2) (Ouyang *et al*., 2014). Briefly, the 3‐UTR of each *SlyAGO* gene was amplified using gene‐specific primers and cloned into the pTRV2 vector (Table S1). Vectors for silencing of the phytoene desaturase (*PDS*) gene were used as a positive control (Ma *et al*., 2015). Four weeks after infiltration of the vector in cotyledons, the transcript levels of the *SlyAGOs* were measured using quantitative real‐time polymerase chain reaction (qRT‐PCR) for individual VIGS plants. The same plants were then infected with *Fol* or water for phenotypic analysis. Disease symptoms of VIGS plants were assessed after a further 2 wk.

### AGO protein immunoprecipitation (IP) and the construction of RNA‐IP libraries

The open reading frames for *SlyAGO4a* and *SlyAGO1* were amplified from tomato cDNA using specific primers and cloned into the pMAL‐c2X vector (New England Biolabs). SlyAGO4a and SlyAGO1 were expressed as N‐terminal maltose binding protein (MBP) fusions in *Escherichia* *coli* strain K12 ER2508 (cat. no. E4127; New England Biolabs), with induction using 300 μM IPTG (isopropyl β‐d‐1‐thiogalactopyranoside; cat. no. 15502; Sigma‐Aldrich) and the fusion protein purified from cell extracts using an amylose resin according to the manufacturer’s recommendations (New England Biolabs). A polyclonal antiserum specific for each fusion protein was raised in rabbits by Cocalico Biologicals, Inc. (Stevens, PA, USA).

Small RNAs were purified by immunopurification of SlyAGO complexes as described (Qi & Mi, 2010). Briefly, 10 g of roots collected at 24 h after infection with *Fol* were ground into a fine powder under liquid nitrogen, and then homogenized in 10 ml of extraction buffer (20 mM Tris‐HCl (pH7.5), 300 mM NaCl, 5 mM MgCl_2_, 5 mM dithiothreitol (DTT), with one tablet of complete EDTA‐free Protease Inhibitor Cocktail (cat. no. 4693132001; Roche). After spinning, the protein lysate was aliquoted into quantities of 20 µl, for use as input samples for Western blot, and 5 µl, for use as a loading control. 10 μl SlyAGO antibody was added to the extract, followed by incubation at 4°C for 4 h. Protein A agarose beads were then added to each sample, and incubation continued for 4 h. After incubation, the protein A beads were collected by spinning and washed three times (10 min each) with 1 ml washing buffer (20 mM Tris‐HCl (pH 7.5), 300 mM NaCl, 5 mM MgCl_2_, 5 mM DTT, 0.5% Triton X‐100, one tablet of complete EDTA‐free Protease Inhibitor Cocktail). The washed beads were resuspended in 200 µl of washing buffer, and then aliquoted into quantities of 20 µl, for use as as pull‐down samples for Western blot, and 5 µl for use as a loading control. For detection of proteins in IP, the washed beads were boiled in 20 µl 2XSDS‐loading buffer and resolved in 12.5% sodium dodecyl sulfate–polyacrylamide gel electrophoresis (SDS‐PAGE) gel. Small RNAs were extracted from the immunoprecipitated SlyAGO complex using the TRIzol reagent and used for small library construction. The libraries were subsequently sequenced and analyzed as described above.

## Results

### *Fol‐*milR1 is exported into tomato cells after host infection

**Fig. 1:**
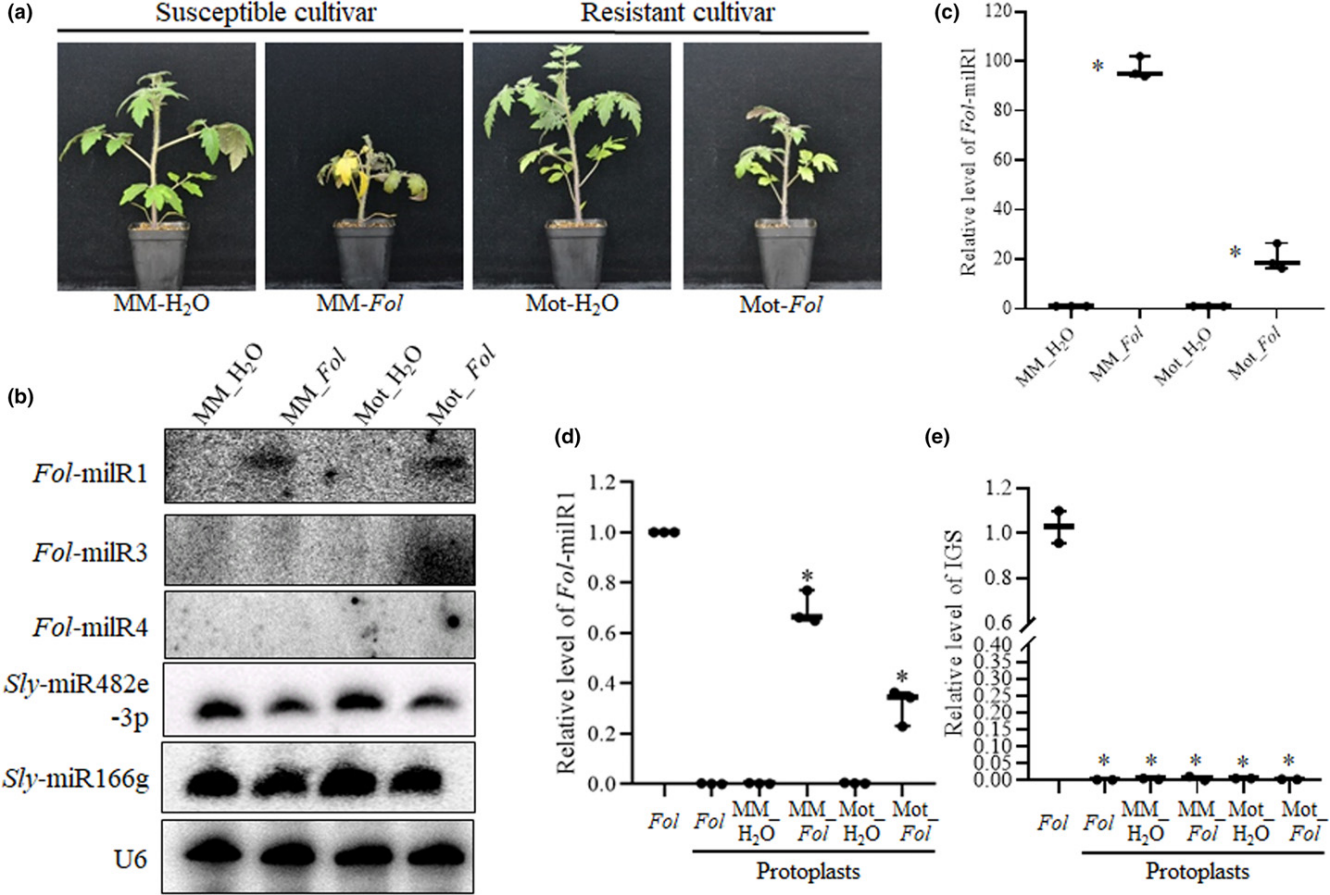
*Fol‐*milR1 is exported into tomato host cells during infection. (a) Tomato wilt disease symptoms caused by infection with *Fol* for 2 wk in susceptible cultivar ‘Moneymaker’ (MM) and resistant cultivar ‘Motelle’ (Mot). (b) Detection of *Fol*‐milR1 in treated tomato roots using low molecular weight RNA gel blots. 40 μg of total RNA was separated by electrophoresis on 8% sodium dodecyl sulphate–polyacrylamide gel electrophoresis (SDS‐PAGE) gels and transferred to a nylon N+ membrane. (γ‐32P)ATP‐labelled specific oligonucleotide probe sequences were used for hybridization. The snRNA gene *U6* was used as a loading control. No hybridization could be detected for *Fol*‐milR3 or *Fol*‐milR4 (shown) or the other four *Fol*‐milRNAs (data not shown). (c) *Fol‐*milR1 expression was confirmed using quantitative real‐time polymerase chain reaction (qRT‐PCR) with specific primers. Asterisks indicate significant difference when compared to the corresponding control plants in the same treatment, according to the Chi‐square test (*, *P* < 0.05). Error bars represent the SD of three replicates. (d) *Fol‐*milR1 was detected in *Fol*, *Fol* protoplasts and tomato root protoplasts using qRT‐PCR with specific primers. Asterisks indicate significant difference when compared to the corresponding control plants in the same treatment, according to the Chi‐square test (*, *P* < 0.05). Error bars represent the SD of three replicates. (e) Relative levels of fungal biomass were presented by ribosomal intergenic spacer region (IGS) amplified from genomic DNA correlates with*Fol* biomass in *Fol*, *Fol* protoplasts and tomato root protoplasts, using quantitative‐PCR with specific primers. Asterisks indicate significant difference when compared to the corresponding control plants in the same treatment, according to a chi‐squared test (*, *P* < 0.05). Error bars represent the SD of three replicates.

This study was initiated by generating four sRNA libraries using roots of the susceptible tomato cultivar ‘Moneymaker’ and the resistant cultivar ‘Motelle’ root with or without infection by *Fusarium oxysporum* f. sp. *lycopersici* (*Fol*) (Fig. 1a). Next generation sequencing (NGS) profiling of sRNAs from these libraries revealed seven novel sRNAs that were identical in sequence to *Fusarium oxysporum* miRNA‐like RNAs (*Fol*‐milRNA), among which *Fol‐*milR1 was the most abundant (Tables 1, S2). Northern blot and qRT‐PCR analysis showed that *Fol‐*milR1 accumulated to readily detectable levels in both infected tomato cultivars (Figs 1b,c, S2). Notably, *Fol‐*milR1 was significantly more abundant in ‘Moneymaker’ than in ‘Motelle’ (Fig. 1b,c). The other candidate miRNAs (*Fol‐*milR2, *Fol‐*milR3, *Fol‐*milR4, *Fol‐*milR5, *Fol‐*milR6 and *Fol‐*milR7) were not detected, presumably due to low abundance (data not shown; Fig. 1b).

**Table 1:** Detection of seven small RNAs from *Fol* in infected tomato plants using RNA‐sequencing.

miR_name	MM_H_2_O	MM_*Fol*	Mot_H_2_O	Mot_*Fol*
*Fol*‐milR1	9	3322	23	1612
*Fol*‐milR2	0	8	0	6
*Fol*‐milR3	0	23	0	0
*Fol*‐milR4	0	15	0	0
*Fol*‐milR5	0	5	0	0
*Fol*‐milR6	0	8	0	0
*Fol*‐milR7	0	10	0	0

MM, Moneymaker; Mot, Motelle.

The presence of a milRNA in the library could result from expression and retention in *Fol* or due to export into, and retention in, the plant host cells. We explored the possibility that *Fol*‐milR1 was exported from *Fol* into tomato cells by generating protoplasts of mock and *Fol*‐infected tomato roots. By utilizing the differences between plant wall components (cellulose, hemicellulase, and pectin) and fungal cell components (chitin and glucans), plant protoplasts were produced by specifically digesting the plant cell wall with cellulase, macerozyme and hemicellulase (Bowman & Free, 2006; Cai & Jin, 2021). Two controls were used to rule out the possible contamination of tomato root protoplasts with *Fol* cells: *Fol* total RNA as a positive control and *Fol* digested with cellulase, macerozyme and hemicellulase as a negative control. We extracted total RNA from tomato root protoplasts, followed by qRT‐PCR analysis. The results indicated that *Fol‐*milR1 was detected in both infected ‘Moneymaker’ and ‘Motelle’ cells, but not in protoplasts from mock‐treated plants (Fig. 1d). We also extracted genomic DNA from digested samples inculding tomato roots and *Fol* mentioned above, and checked the relative levels of fungal ribosomal intergenic spacer region (IGS) amplified from genomic DNA correlates with *Fol* biomass in *Fol*, *Fol* protoplasts or tomato root protoplasts, respectively, using qPCR with specific primers. The results showed that *Fol* biomass was only detected in *Fol*, but not in either *Fol* protoplasts or tomato protoplasts (Fig. 1e). These findings are consistent with export of *Fol‐*milR1 into the host plant cell after infection with the pathogen.

### *Fol‐*milR1 is essential for the pathogenicity of *Fol*

**Fig. 2:**
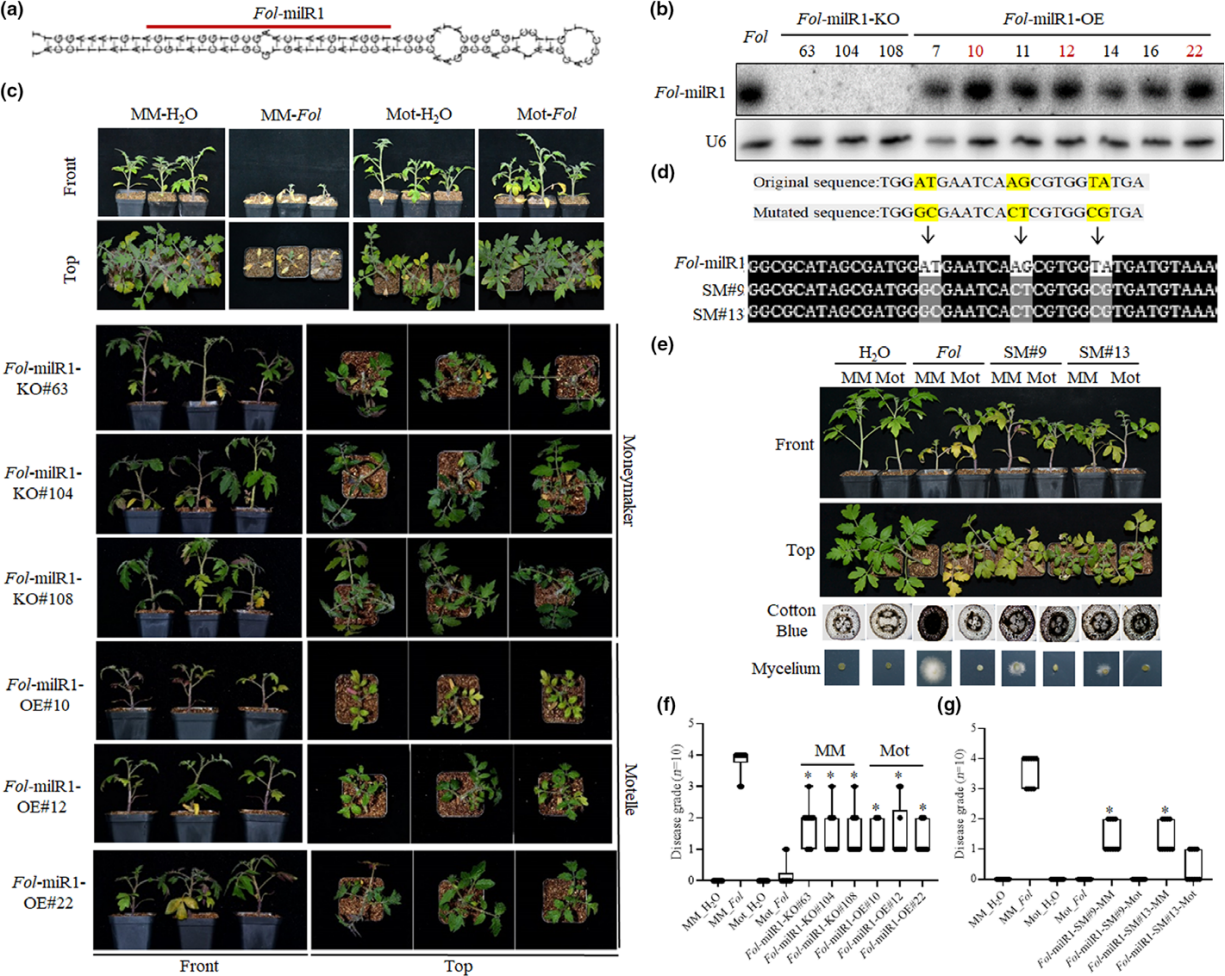
*Fol‐*milR1 is essential for *Fol* pathogenicity. (a) The pre‐miRNA‐like stem‐loop structure of precursor *Fol‐*milR1. (b) Identification of *Fol‐*milR1‐KO (knockout) and *Fol‐*milR1‐OE (overexpression) strains using low molecular weight RNA gel blots. Three *Fol‐*milR1‐KO and three *Fol‐*milR1‐OE strains (highlighted in red) were recruited for subsequent experiments. (c) The *Fol‐*milR1‐KO and *Fol‐*milR1‐OE strains and control wild‐type *Fol* were used to inoculate tomato seedlings. Wilt disease symptoms were photographed 2 wk after inoculation. (d) Generation of *Fol‐*milR1 site‐mutated strains. Mutated sites were highlighted in yellow. The mutated sites were confirmed by sequencing. (e) The *Fol‐*milR1‐site‐mutated strains and control wild‐type *Fol* were used to inoculate tomato seedlings. Wilt disease symptoms were photographed 2 wk after inoculation. Cotton blue staining results reflect the abundance of *Fol* in the stem of tomato plants. More intense cotton blue staining correlates with higher levels of *Fol*. (f) Disease grades for all pathogen infection assays at 14 d post inoculation (dpi). The asterisks indicate significant differences in the wilt disease symptoms of *Fol‐*milR1‐KO strains vs wild‐type *Fol* in ‘Moneymaker’ (MM), and *Fol‐*milR1‐OE strains vs wild‐type *Fol* in ‘Motelle’ (Mot) according to the Chi‐square test (*, *P* < 0.05). Error bars represent the SD of three replicates. (g) Disease grades for *Fol* infection assays at 14 dpi. The asterisks indicate significant differences in the wilt disease symptoms of *Fol‐*milR1‐SM strains vs wild‐type *Fol* in ‘Moneymaker’ according to the Chi‐square test (*, *P* < 0.05). Error bars represent the SD of three replicates.

As an sRNA that is 23 nucleotides (nts) in length, *Fol‐*milR1 is unique, and it is derived from a pre‐miRNA‐like stem‐loop structure of canonical appearance, in which the *Fol‐*milR1 sequence occupies one side of the predicted stem (Fig. 2a). To assess whether *Fol‐*milR1 is required for pathogenesis of *Fol*, we used a gene replacement construct strategy to delete (Fig. S3) or overexpress *Fol‐*milR1. Three knockout mutants (*Fol‐*milR1‐KO#63, #104 and #108) and several overexpression strains (*Fol‐*milR1‐OE#7, #10, #11, #12, #14, #16 and #22) were confirmed by Northern blot (Fig. 2b). The relative levels of *Fol‐*milR1 in *Fol‐*milR1‐KO#63, #104 and #108 strains and *Fol‐*milR1‐OE_#10, #12 and #22 strains were also checked using stem‐loop qRT‐PCR (Fig. S4) and carried forward for experiments. Knockout or overexpression of *Fol‐*milR1 did not significantly alter growth or colony morphology of *Fol* under a variety of stress conditions (Fig. S5). The KO, OE and SM mutant lines of *Fol* did not impair spore production (data not shown). The six strains were used to infect the resistant cultivar ‘Motelle’ and the susceptible cultivar ‘Moneymaker’. The *Fol‐*milR1‐KO#63, #104 and #108 knockout strains exhibited attenuated pathogenesis in ‘Moneymaker’ seedlings, supported by observations of a lower grade of wilt disease symptoms compared to wild‐type *Fol*‐treated ‘Moneymaker’ (Fig. 2c,f). By contrast, *Fol‐*milR1‐OE#10, #12 and #22 overexpression strains caused obvious wilt disease symptoms in ‘Motelle’ plants, supported by observations of a higher grade of wilt disease symptoms compared to wild‐type *Fol*‐treated ‘Motelle’ (Fig. 2c,f). These results are consistent with a positive role for *Fol*‐milR1 in pathogenesis towards tomato.

To further evaluate the pathogenicity of *Fol‐*milR1, we generated site‐mutated *Fol* strains *Fol‐*milR1‐SM#9 and *Fol‐*milR1‐SM#13 (Fig. 2d). The introduced mutations were confirmed by sequencing. After inoculation of ‘Motelle’ and ‘Moneymaker’ with wild‐type *Fol* and both mutated strains, we observed impaired infection of the susceptible ‘Moneymaker’ supported by staining for the presence of the fungus within the plant stem, fungal mycelium regeneration and lower wilt disease grade compared to wilt pathogen treated ‘Moneymaker’, while ‘Motelle’ was unchanged relative to infection with wild‐type *Fol* (no disease symptoms). These findings correlate with those obtained for the *Fol*‐milR1 knockout mutants *Fol*‐milR1‐KO#63, #104 and #108 (Fig. 2e,g). Based on these results, we conclude that *Fol‐*milR1 is a critical pathogenic factor responsible for impaired tomato defense against *Fol*.

### *Fol‐*milR1 regulates the *SlyFRG4* target gene in host plants

**Fig. 3:**
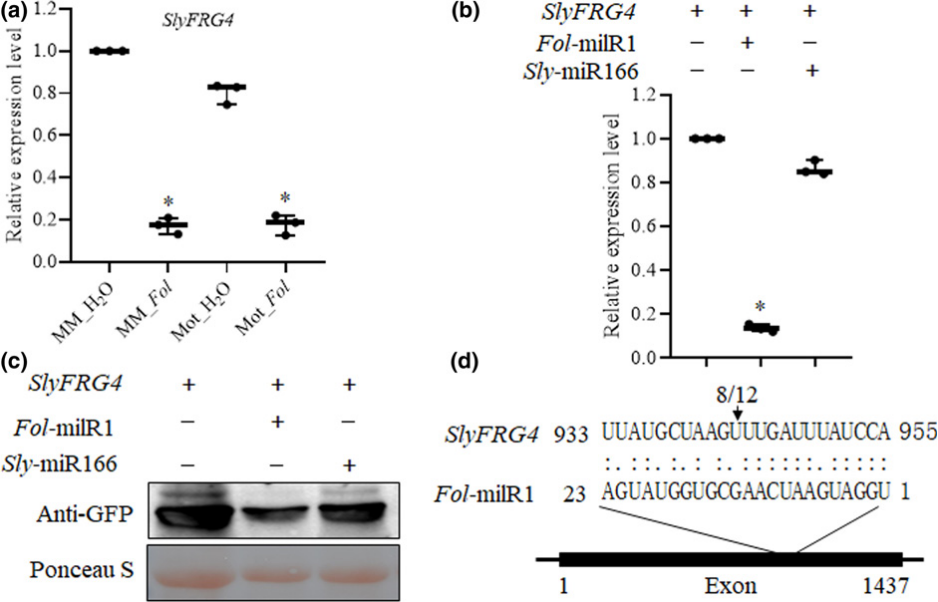
*Fol‐*milR1 regulates *SlyFRG4* expression at the posttranscriptional level. (a) *SlyFRG4* mRNA levels are repressed after *Fol* infection in both ‘Moneymaker’ (MM) and ‘Motelle’ (Mot). Asterisks indicate significant difference when compared to the corresponding control plants in the same treatment, according to the Chi‐square test (*, *P* < 0.05). Error bars represent the SD of three replicates. (b) Level of *SlyFRG4* target mRNA during co‐infiltration experiments in *Nicotiana benthamiana*. Quantitative real‐time polymerase chain reaction (qRT‐PCR) was used to determine the relative levels of *SlyFRG4* in *N. benthamiana* leaves expressing *SlyFRG4* only, *SlyFRG4* + *Fol‐*milR1 or *SlyFRG4* + control miRNA (*Sly‐*miR166). Values were normalized to *N. benthamiana* actin. Asterisks indicate significant difference when compared to the corresponding control plants in the same treatment, according to the Chi‐square test (*, *P* < 0.05). Error bars represent the SD of three replicates. (c) SlyFRG4‐GFP fusion protein was detected by Western blot using anti‐GFP antibody. Crude protein extracts prepared from *N. benthamiana* leaves in (b) were electrophoresed on sodium dodecyl sulfate–polyacrylamide gel electrophoresis (SDS‐PAGE) gels and blotted onto nitrocellulose membranes (top panel). A duplicate gel was Ponceau S‐stained as a loading control (bottom panel). A minimum of 10 individual leaf samples were used for each experiment. (d) The cleavage site in the *SlyFRG4* mRNA was determined using 5′RLM‐RACE. The arrow indicates the 5′ terminus of miRNA‐guided cleavage products and the frequency of clones (8/12) is shown. The cDNA of *SlyFRG4* contains one single large exon.

We next combined computational prediction (psRNATarget algorithm (Dai *et al*., 2018)) with *Agrobacterium*‐mediated transient co‐expression experiments in *N. benthamiana* to identify the tomato genes targeted by the imported *Fol‐*milR1 (Table S3). Among the predicted genes, *Solyc06g007430* (encoding a CBL‐interacting protein kinase, termed *Fusarium* resistance gene 4, *SlyFRG4*) was regulated at the transcriptional level in both ‘Moneymaker’ and ‘Motelle’ after *Fol* infection (Fig. 3a). To determine whether *Fol‐*milR1 regulates *SlyFRG4* expression, we conducted an *Agrobacterium*‐mediated transient co‐expression experiment in *N. benthamiana* (Ouyang *et al*., 2014). Quantitative real‐time polymerase chain reaction results showed that the mRNA level of *SlyFRG4* was significantly reduced in the presence of *Fol‐*milR1 (Fig. 3b). Western blot assays using an anti‐GFP antibody demonstrated that SlyFRG4 protein was greatly downregulated in the presence of *Fol‐*milR1 (Fig. 3c). Green fluorescent protein was not detected in control infiltration experiments of *Agrobacterium* with the empty vector and the p19‐construct alone, respectively (data not shown). These results are consistent with *Fol‐*milR1 acting to decrease levels of the *SlyFRG4* transcript. 5’‐RNA ligase‐mediated rapid amplification of cDNA ends (5′RLM‐RACE) analysis further showed the mRNA cleavage site occurred at nucleotide 943 of the *SlyFRG4* coding region in 8 out of 12 clones (Figs 3d, S6 shows sequencing for clones highlighted in red). Together with our previous findings, these results support a scenario in which *Fol‐*milR1 is exported into tomato cells to silence the host gene *SlyFRG4*.

### *SlyFRG4* is required for wilt disease resistance in tomato

**Fig. 4:**
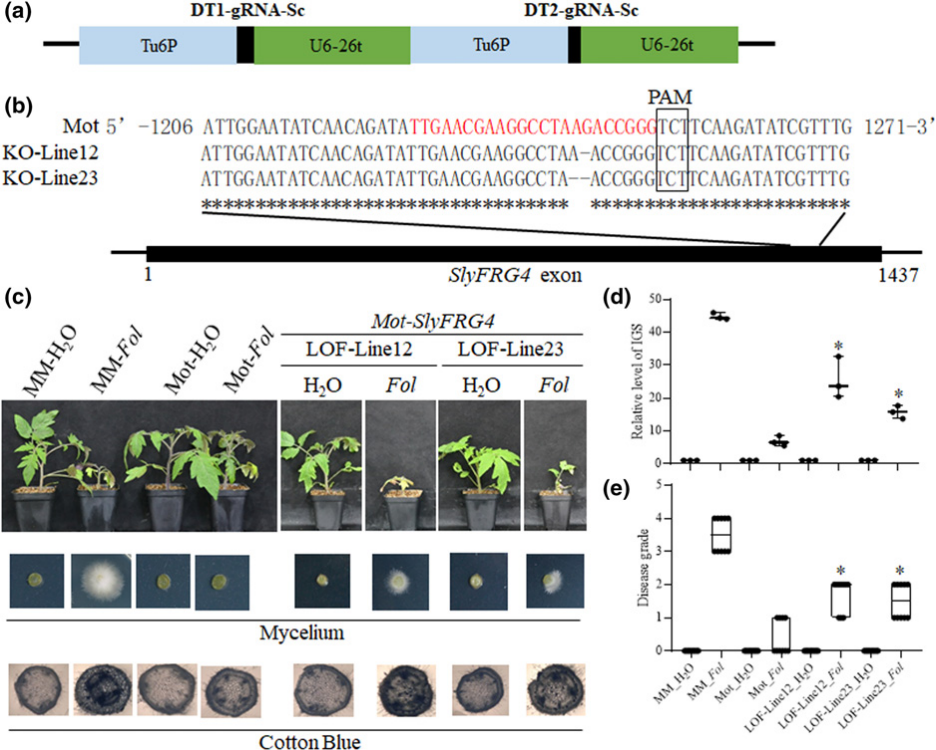
*SlyFRG4* is required for *Fol* resistance. (a) Schematic diagram of the CRISPR/Cas9 cassette used for mutation of *SlyFRG4*. (b) clustalx nucleic acid sequence alignments of genomic sequences obtained for *SlyFRG4*‐LOF plants. The sequence of the gRNA is highlighted with red. (c) Loss of function (LOF) of *SlyFRG4* attenuates the resistance to *Fol* in ‘Motelle’. Cotton blue staining results reflect the abundance of *Fol* in the stem of tomato plants. More intense cotton blue staining correlates with greater abundance of *Fol*. (d) Relative levels of fungal ribosomal intergenic spacer region (IGS) amplified from genomic DNA correlates with *Fol* biomass in tomato plants at 2 wk after inoculation with *Fol*. The asterisks indicate significant differences in the *Fol* biomass of *SlyFRG4* loss‐of‐function alleles vs ‘Motelle’ after *Fol* infection according to the Chi‐square test (*, *P* < 0.05). Error bars represent the SD of three replicates. (e) Disease grades for *Fol* infection assays at 14 d post inoculation (dpi). The asterisks indicate significant differences in the wilt disease symptoms of *SlyFRG4*‐LOF alleles vs ‘Motelle’ after *Fol* infection according to the Chi‐square test (*, *P* < 0.05). Error bars represent the SD of three replicates. KO, knockout; MM, cv Moneymaker; Mot, cv Motelle; PAM, protospacer adjacent motif.

We hypothesize that *Fol* exports miRNAs to facilitate fungal pathogenesis and achieve plant colonization. To characterize the functions of *SlyFRG4* in response to *Fol* infection, we generated CRISPR/Cas9 *SlyFRG4* loss‐of‐function (LOF) alleles in the resistant ‘Motelle’ cultivar (Deng *et al*., 2018) (Fig. 4a). Two transgenic plants, termed *SlyFRG4*‐KO‐Line 12 and 23, carrying 1‐ and 2‐nucleotide deletions respectively, were identified (Fig. 4b). We then inoculated the *SlyFRG4*‐LOF‐allele lines and ‘Motelle’ and ‘Moneymaker’ control plants with *Fol*. Both of the *SlyFRG4*‐LOF‐allele lines exhibited severe wilt symptoms in leaves relative to resistant cultivar ‘Motelle’, while presenting phenotypes similar to the treated ‘Moneymaker’ seedlings (Fig. 4c–e). *SlyFRG4*‐LOF alleles also accumulated more *Fol* biomass and higher wilt disease symptom than ‘Motelle’ controls. These results further confirm that *SlyFRG4* is essential for resistance to tomato wilt disease.

### *Fol‐*milR1 mediates host immunity through association with SlyAGO4a

It has been well documented that ARGONAUTE proteins (AGOs) mediate small‐RNA‐induced‐gene‐silencing events by forming a core constituent of the silencing effector RISC interacting with various partners, such as Dicer, TRBP and GW182 family proteins (Carmell *et al*., 2002; Baumberger & Baulcombe, 2005; Chen *et al*., 2014). *Botrytis* *cinerea* sRNAs hijack the host RNAi machinery by binding to Arabidopsis AGO1 to mediate host immunity (Weiberg *et al*., 2013).

To further investigate whether SlyAGOs are essential for tomato wilt disease, we utilized the VIGS approach to knock down each predicted *SlyAGO* gene in the tomato genome in the susceptible cultivar ‘Moneymaker’. After inoculating with *Fol*, VIGS‐*SlyAGO4a* plants showed the greatest decrease in disease susceptibility of all VIGS‐SlyAGO plants tested (Table S4; Fig. S7).

We have shown that *Fol‐*milR1 is a novel sRNA effector that is 23 nts in length. This sRNA structure is proposed to serve as the passenger strand for the 24 nt guide siRNAs that become stably associated with AGO4 in Arabidopsis (Singh *et al*., 2019). SlyAGO4a shares 74.8% amino acid identity with Arabidopsis AtAGO4 (Fig. S8).

**Fig. 5:**
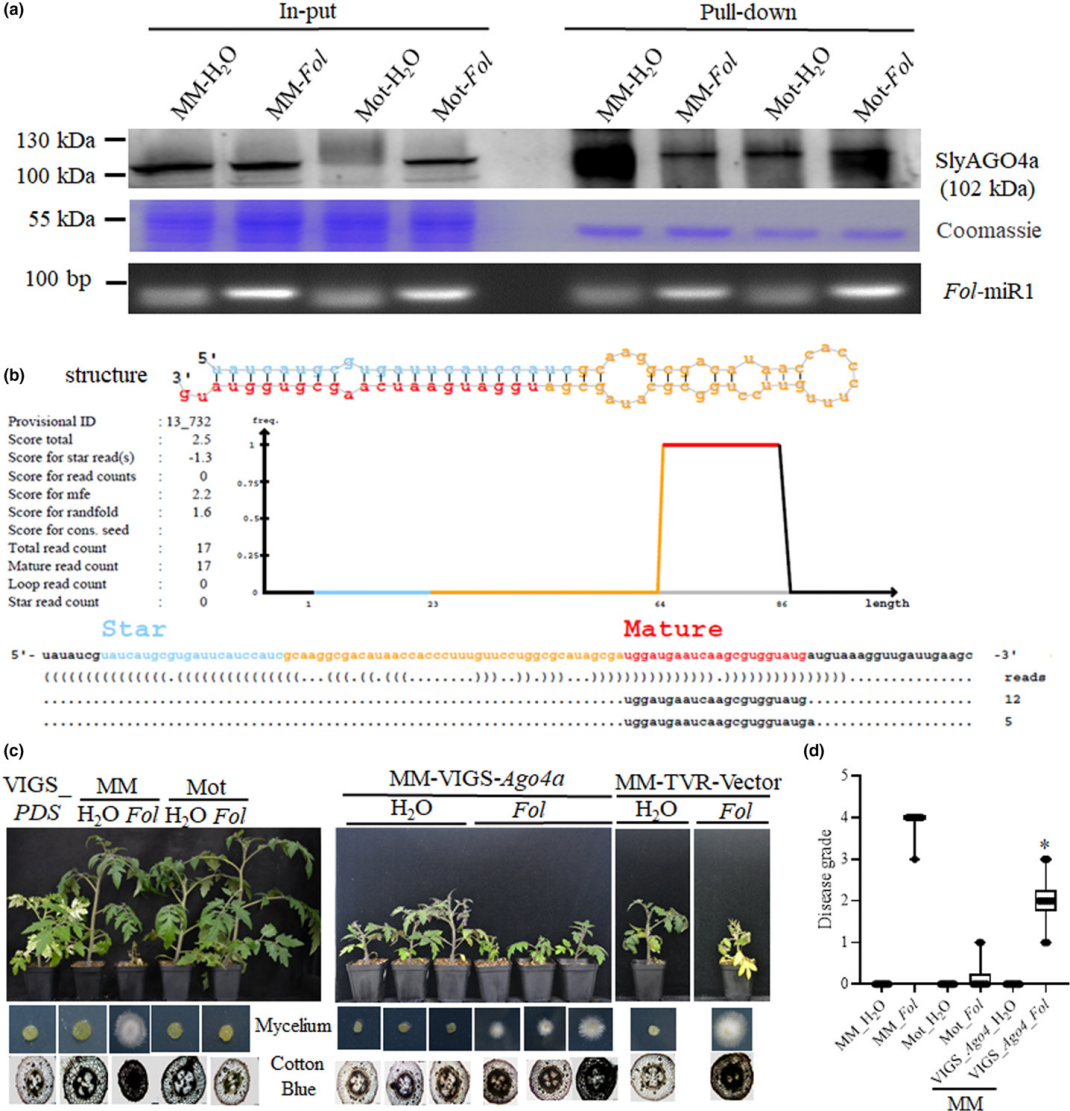
*Fol‐*milR1 associates with SlyAGO4a to suppress host immunity. (a) Association of *Fol‐*milR1 with SlyAGO4a during infection. SlyAGO4a was immunoprecipitated (IP) from *Fol*‐infected roots harvested at 24 h after inoculation using a SlyAGO4a polyclonal antibody. Total RNA was extracted from the SlyAGO4a‐IP fraction and used for stem‐loop quantitative real‐time polymerase chain reaction (qRT‐PCR). (b) Novel‐m0003‐3p, homologous to *Fol‐*milR1, was detected in the SlyAGO4a‐IP sample using sRNA‐sequencing. (c) *SlyAGO4a*‐VIGS plants exhibit reduced disease susceptibility to *Fol* compared with the susceptible ‘Moneymaker’. In total, 30 *SlyAGO4a*‐VIGS plants were generated, and 25 exhibited reduced disease susceptibility to *Fol*. *phytoene desaturase* (*PDS*). TRV‐silenced plants (TRV‐*PDS*) and TRV‐vector plants were used as positive controls for silencing. (d) Disease grades for *Fol* infection assays at 14 dpi. The asterisks indicate significant differences of the wilt disease symptoms of *SlyAGO4a*‐VIGS plants vs ‘Moneymaker’ after *Fol* infection according to the Chi‐square test (*, *P* < 0.05). Error bars represent the SD of three replicates. MM, cv Moneymaker; Mot, cv Motelle; PDS, phytoene desaturase; VIGS, virus‐induced gene silencing.

To test our hypothesis that *Fol‐*milR1 associates with SlyAGO4a in tomato, we first overexpressed and purified the SlyAGO4a and SlyAGO1 proteins from *E. coli* and then used the purified proteins to generate a polyclonal antiserum in rabbits (see ‘AGO protein immunoprecipitation (IP) and the construction of RNA‐IP libraries’ in the Materials and Methods section). The antisera reacted with species of the predicted sizes of the two proteins in extracts from tomato leaves and roots (Fig. S9a). We determined that SlyAGO4a did not bind to any potential *Fol* AGO proteins, in spite of possessing the highly conserved PAZ and PIWI domains found in fungal AGO proteins (Fig. S9a) (Fang & Qi, 2016). To determine whether *Fol‐*milR1 can associate with tomato AGO4a or AGO1, we performed immunoprecipitation (IP) experiments using each polyclonal antibody with extracts prepared from *Fol*‐infected tomato roots collected at 24 h after inoculation (Fig. 5a). Total RNAs were extracted from the immunoprecipitates, followed by qRT‐PCR using *Fol‐*milR1 specific primers. *Fol‐*milR1 was clearly detected in the SlyAGO4a‐associated fraction from both infected ‘Moneymaker’ and ‘Motelle’ samples (Fig. 5a), but only faint bands were visible in mock or SlyAGO1‐associated fractions (Fig. S9b). We further constructed two sRNA libraries using material immunoprecipitated from extracts prepared from ‘Moneymaker’ and ‘Motelle’ infected with *Fol* using the SlyAGO4a polyclonal antiserum. A miRNA termed novel‐m0003‐3p, corresponding to *Fol‐*milR1, was detected in the SlyAGO4a‐IP sample (Fig. 5b; Tables 2, S5). These results support a physical interaction between *Fol‐*milR1 and AGO4a in tomato.

**Table 2:** Detection of novel small RNAs from the SlyAGO4a‐IP sample using sRNA‐sequencing.

Mature_ id	Hairpin_id	Genomic_id	Hairpin_start	Hairpin_end	Hairpin_strand	Hairpin_length (nt)	miRDeep2_score	Hairpin_GC	Mature_seq	Mature_Length (nt)	Hairpin_seq	Hairpin_struct
novel‐m0001‐5p	novel‐m0001	5	2608121	2608162	+	42	0	58.54	CACGTTTCCTGCTGACCT	18	CACGTTTCCTGCTGACCTTCATGGTCAAGCAGCGGCTCCGT	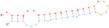
novel‐m0002‐5p	novel‐m0002	9	2235106	2235183	+	78	2	51.95	TCTGCTGACTCACGTTTCGATGGGTTCTTCGG	24	TCTGCTGACTGATGGGTTCTTCGGAGTACTTGAGGCTTGCCTTGAGGACAGTGTAGAACTTTTGAGGTCAGTGGGGG	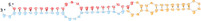
novel‐m0003‐3p	novel‐m0003	13	1478130	1478215	–	86	2.5	49.41	TGGATGAATCAAGCGTGGTATG	22	TATCATGCGTGATTCATCCATCGCAAGGCGACATAACCACCCTTTGTTCCTGGCGCATAGCGATGGATGAATCAAGCGTGGTATG	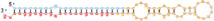
novel‐m0004‐3p	novel‐m0004	10	843193	843241	–	49	10	75	AGGGGGGAGCGCAACTGGAGC	21	TCTGGTTGCGCCCCCCTCTGGGCCCCCAGGGGGGAGCGCAACTGGAGC	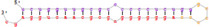

Subsequent analysis of the VIGS‐*SlyAGO4a* tomato seedlings showed that they displayed significantly greater disease resistance in response to *Fol* relative to the susceptible ‘Moneymaker’ cultivar (Fig. 5c). The infected VIGS‐*SlyAGO4a* tomato seedlings showed a lower grade of wilt disease symptoms relative to the susceptible cultivar ‘Moneymaker’ (Fig. 5d). In total, 30 *SlyAGO4a*‐VIGS plants were generated, and 25 of them exhibited reduced disease susceptibility to *Fol*. This result shows that SlyAGO4a is required for the susceptibility of ‘Moneymaker’ towards *Fol*. Taken together, these findings provide support for a mechanism in which the enhanced resistance to *Fol* observed in *SlyAGO4a*‐VIGS ‘Moneymaker’ plants results from reduced association of *Fol‐*milR1 with SlyAGO4a, thus blocking *Fol‐*milR1 processing and the suppression of host immunity during early infection.

## Discussion

Plant pathogenic fungi such as *B*. *cinerea*, *Magnaporthe oryzae* and *Fusarium graminearum* have been extensively studied as model pathosystems to explore the molecular mechanism of pathogen–host interactions (Dean *et al*., 2012). To achieve successful host colonization, pathogens transmit many types of macromolecules, the most prominent being effector proteins, to suppress host innate immunity (Mendgen & Hahn, 2002; Kim & Westwood, 2015). While protein effector‐triggered immunity in pathogen–host interactions has been well studied (Bigeard *et al*., 2015; Li *et al*., 2016), the mechanisms underlying trans‐kingdom sRNA‐mediated plant immunity remain elusive.

In this study, we identified a novel virulence strategy for *Fol* to achieve plant infection by exporting *Fol‐*milR1 as an sRNA effector to silence a specific host resistance gene and suppress host immunity. So far, only a few sRNAs, such as *Bc*‐siRNAs from *B*. *cinerea* (Weiberg *et al*., 2013; M. Wang *et al*., 2017), *Pst*‐milR1 from *P*. *striiformis* (B. Wang *et al*., 2017), and *ccm*‐miRNAs from *C. campestris* (Shahid *et al*., 2018), were reported to be exported from pathogens or parasitic plants to host plants during infection. Silenced *Pst*‐milR1 using the host‐induced gene silencing (HIGS) system in the wheat cultivar ‘Su11’ reduces the virulence of *P*. *striiformis* (B. Wang *et al*., 2017). However, Arabidopsis plants ectopically expressing *Bc*‐siRNAs using a plant artificial miRNA vector display enhanced susceptibility to *B. cinerea* (Weiberg *et al*., 2013). Our data showed that knockout or site‐mutation of *Fol‐*milR1 attenuates the pathogenicity of *Fol*, while overexpression of *Fol‐*milR1 enhances the virulence of *Fol*, leading to obvious wilt disease symptom in the resistant cultivar ‘Motelle’. Therefore, we concluded that *Fol‐*milR1 may function as a critical sRNA effector by contributing directly to pathogenicity to overcome host defense responses.

It is well known that miRNAs are a class of negative post‐transcriptional regulators of their target genes. The trans‐kingdom sRNAs from pathogens or parasitic plants target the coding regions of resistant genes in their hosts. Intriguingly, our data demonstrated that *Fol‐*milR1 regulates the expression of host wilt disease resistance gene *SlyFRG4* at the posttranscriptional level. Previously, tomato endogenous miRNAs miR482e‐3p and miR5300 were reported to be repressed under *Fol* infection, leading to increased expressions of several targets genes encoding nucleotide‐binding site and leucine‐rich repeat domain containing proteins (*NBS‐LRR*s), which are essential disease resistance genes in plants (Ouyang *et al*., 2014; Ji *et al*., 2018b; Gao *et al*., 2020). Our results show that *SlyFRG4* was suppressed by *Fol* infection in both cultivars (Fig. 3a). We further verified that *Fol*‐milR1 targeted *SlyFRG4* by the transient co‐expression experiments in *N. benthamiana* (Fig. 3b–d). The *SlyFRG4* loss‐of‐function alleles in resistant cultivar ‘Motelle’ displayed relative susceptible wilt disease symptoms (Fig. 4c–e). Hence, we speculated that the expression level of *SlyFRG4* was suppressed by trans‐kingdom *Fol*‐milR1 upon the inversion of *Fol*, which impaired the resistance in tomato. These results coherently demonstrate that *SlyFRG4* is essential for resistance to tomato wilt disease. Since the efficient transmission of natural pathogen RNAi triggers have been explored, we propose that the export of small silencing RNAs to downregulate the wilt disease resistant gene expression in the host represents a conserved pathogen infection strategy to combat host defense.

sRNAs regulate target gene expression by binding to AGO clade proteins based on the sequence specificity following the 5′ nucleotide‐directed loading rule (Wu *et al*., 2009; Fang & Qi, 2016). All sRNAs mentioned above are 21 nt in length. At 23 nt in length, *Fol‐*milR1 differs from these reported 21 nt sRNAs. sRNAs from plant hosts have been recognized as regulators of host–microbial interactions (Ruiz‐Ferrer & Voinnet, 2009; Wu *et al*., 2009; Weiberg *et al*., 2013; Fang & Qi, 2016; Zhang *et al*., 2016; B. Wang *et al*., 2017; Cai *et al*., 2018; Shahid *et al*., 2018). *Bc*‐sRNAs, the first reported sRNA effectors in *B. cinerea*, are exported into Arabidopsis and bind to AGO1, leading to suppression of host immunity (Weiberg *et al*., 2013). Using a polyclonal antibody specific to SlyAGO4a, our results suggest that *Fol‐*milR1 associates with SlyAGO4a to reduce plant immunity, leading to effective infection, which is an AGO1‐associated independent invasion strategy. Moreover, exported *Bc*‐siRNAs are detected in *B. cinerea dcl1* or *dcl2* single mutants, but not in a *dcl1dcl2* double mutant, with significantly reduced fungal virulence (Weiberg *et al*., 2013). In a subsequent study, transgenic tomato and Arabidopsis expressing sRNAs targeting *B. cinerea DCL* transcripts display resistance to pathogen infection (Wang *et al*., 2016). Recently, *Hpa*‐siRNAs from the pathogen *Hyaloperonospora arabidopsidis* were found to employ the host Arabidopsis's Argonaute 1 (*At*AGO1)/RNA‐induced silencing complex for virulence. Furthermore, a novel CRISPR endoribonuclease Csy4/GUS reporter was developed to visualize *Hpa*‐siRNA‐induced target suppression in Arabidopsis *in situ* (Dunker *et al*., 2020).

So far, several AGO proteins have been identified in Arabidopsis, among them, AGO1 predominates in the miRNA pathway (Morel *et al*., 2002; Qi *et al*., 2005), and AGO4 plays redundant roles in repeat associated siRNA (rasiRNA) accumulation and DNA methylation, as well as transcriptional gene silencing (TGS) at specific genomic loci (Zilberman *et al*., 2003; Qi *et al*., 2006). Here, we report that *Fol*‐milR1 downregulated *SlyFRG4* levels after being transferred into the host plant. We do not have evidence that *Fol*‐milR1 directly associates with SlyAGO4a to induce transcriptional gene silencing of *SlyFRG4* in *N. benthamiana*. However, our observation of *Fol*‐milR1 associating with SlyAGO4a, but not with SlyAGO1, using sRNA‐IP in tomato, leads us to propose the involvement of *Fol*‐milR1 in at least two different sRNA silencing pathways. On the other hand, there is no guarantee that findings from a transient heterologous expression experiment hold true in the natural system; it is possible that *Fol*‐milR1 is loaded onto a different AGO during transient expression in *N. benthamiana* than in tomato.

Intriguingly, the 21 nt sRNAs with a 5′U are preferred by AGO1, whereas those with a 5′A are associated with AGO2. By contrast, 24 nt sRNAs with a 5′A are preferentially loaded onto AGO4, whereas AGO5 shows a bias towards sRNAs with a 5′C without an obvious size preference (Mallory & Vaucheret, 2006; Vaucheret, 2006). However, the 5′‐terminal nt‐directed sorting model is not the only mechanism for sRNA sorting onto the AGOs and additional mechanisms are still largely unknown. This last point may be relevant to *Fol*‐milR1, in that it contains a 5′U (Fig. 3), but interacts with AGO4.

This study showed that *Fol*‐milR1 is, uniquely, 23 nt in length and acts as an effector to debilitate plant immunity and achieve infection. To determine whether transmission of *Fol‐*milR1 into tomato causes DNA methylation in the tomato genome and elucidate other aspects of the molecular mechanism involving the action of *Fol*‐milR1, further exploration is required. We propose that export of sRNA to the host plant represents a sophisticated and conserved coevolution between pathogen and host.

## Author contributions

S‐QO designed the experiments, supervised the project and wrote the manuscript. KAB contributed to the design of this project, supervised AGO antibody generation and revised the manuscript. H‐MJ and H‐YM performed the experiments in cooperation with SJ Li, TF, Z‐YZ, LC, and SJ Luo contributed to data analysis and interpretation. All authors read and approved the final manuscript. H‐MJ and H‐YM contributed equally to this work.

## Supporting information


**Fig. S1** The grades of wilt disease severity.
**Fig. S2** The abundance of *Fol* increases with time in both tomato cultivars, but to a much greater extent in ‘Moneymaker’.
**Fig. S3** Targeted gene replacement of *Fol‐*milR1 in *Fol*.
**Fig. S4** *Fol‐*milR1 expression levels in knockout and overexpression transformants, as measured using stem‐loop quantitative real‐time polymerase chain reaction (qRT‐PCR).
**Fig. S5** Growth and colonial morphology of the *Fol‐*milR1‐KO and *Fol‐*milR1‐OE strains in response to various stressors.
**Fig. S6** The 5′RLM‐RACE polymerase chain reaction products used for sequencing.
**Fig. S7** Wilt disease symptoms of VIGS‐*SlyAGOs* and control plants infected by *Fol*.
**Fig. S8** Comparison of homologous regions in SlyAGO4a and AtAGO4.
**Fig. S9** No association can be detected between *Fol‐*milR1 and SlyAGO1.

**Table S1** Primers used in this study.

**Table S2** Summary of reads after RNA‐sequencing of the four libraries.

**Table S3** Predicted targets of *Fol‐*milR1 in the tomato genome.

**Table S4** Statistic of tomato wilt disease of virus‐induced gene silenced (VIGS)‐*SlyAGO*s.

**Table S5** Detection of all small RNAs from SlyAGO4a‐IP samples using sRNA‐sequencing.
Please note: Wiley Blackwell are not responsible for the content or functionality of any Supporting Information supplied by the authors. Any queries (other than missing material) should be directed to the *New Phytologist* Central Office.

## Data Availability

The raw sequence data for this study are available in the GenBank Nucleotide Sequence Databases with accession nos. PRJNA723916 and PRJNA723757.

## References

[nph17436-bib-0001] Asai S , Shirasu K . 2015. Plant cells under siege: plant immune system versus pathogen effectors. Current Opinion in Plant Biology 28: 1–8. 26343014 10.1016/j.pbi.2015.08.008

[nph17436-bib-0002] Atkinson NJ , Urwin PE . 2012. The interaction of plant biotic and abiotic stresses: from genes to the field. Journal of Experimental Botany 63: 3523–3543. 22467407 10.1093/jxb/ers100

[nph17436-bib-0003] Baldrich P , San Segundo B . 2016. MicroRNAs in rice innate immunity. Rice 9: 6. 26897721 10.1186/s12284-016-0078-5 PMC4761359

[nph17436-bib-0004] Baulcombe D . 2004. RNA silencing in plants. Nature 431: 356–363. 15372043 10.1038/nature02874

[nph17436-bib-0005] Baumberger N , Baulcombe DC . 2005. Arabidopsis ARGONAUTE1 is an RNA Slicer that selectively recruits microRNAs and short interfering RNAs. Proceedings of the National Academy of Sciences, USA 102: 11928–11933. 10.1073/pnas.0505461102 PMC1182554 16081530

[nph17436-bib-0006] Bhattacharjee S , Zamora A , Azhar MT , Sacco MA , Lambert LH , Moffett P . 2009. Virus resistance induced by NB‐LRR proteins involves Argonaute4‐dependent translational control. The Plant Journal 58: 940–951. 19220787 10.1111/j.1365-313X.2009.03832.x

[nph17436-bib-0007] Bigeard J , Colcombet J , Hirt H . 2015. Signaling mechanisms in pattern‐triggered immunity (PTI). Molecular Plant 8: 521–539. 25744358 10.1016/j.molp.2014.12.022

[nph17436-bib-0008] Boller T , Felix G . 2009. A renaissance of elicitors: perception of microbe‐associated molecular patterns and danger signals by pattern‐recognition receptors. Annual Review of Plant Biology 60: 379–406. 10.1146/annurev.arplant.57.032905.105346 19400727

[nph17436-bib-0009] Boller T , He SY . 2009. Innate immunity in plants: an arms race between pattern recognition receptors in plants and effectors in microbial pathogens. Science 324: 742–744. 19423812 10.1126/science.1171647 PMC2729760

[nph17436-bib-0010] Bowman SM , Free SJ . 2006. The structure and synthesis of the fungal cell wall. BioEssays 28: 799–808. 16927300 10.1002/bies.20441

[nph17436-bib-0011] Cai Q , Jin H . 2021. Small RNA extraction and quantification of isolated fungal cells from plant tissue by the sequential protoplastation. Methods in Molecular Biology 2170: 219–229. 32797462 10.1007/978-1-0716-0743-5_16

[nph17436-bib-0012] Cai Q , Qiao L , Wang M , He B , Lin FM , Palmquist J , Huang SD , Jin H . 2018. Plants send small RNAs in extracellular vesicles to fungal pathogen to silence virulence genes. Science 360: 1126–1129. 29773668 10.1126/science.aar4142 PMC6442475

[nph17436-bib-0013] Carmell MA , Xuan Z , Zhang MQ , Hannon GJ . 2002. The Argonaute family: tentacles that reach into RNAi, developmental control, stem cell maintenance, and tumorigenesis. Genes & Development 16: 2733–2742. 12414724 10.1101/gad.1026102

[nph17436-bib-0014] Chen CF , Ridzon DA , Broomer AJ , Zhou ZH , Lee DH , Nguyen JT , Barbisin M , Xu NL , Mahuvakar VR , Andersen MR *et al*. 2005. Real‐time quantification of microRNAs by stem‐loop RT‐PCR. Nucleic Acids Research 33: e179. 16314309 10.1093/nar/gni178 PMC1292995

[nph17436-bib-0015] Chen R , Jiang N , Jiang Q , Sun X , Wang Y , Zhang H , Hu Z . 2014. Exploring microRNA‐like small RNAs in the filamentous fungus *Fusarium oxysporum* . PLoS ONE 9: e104956. 25141304 10.1371/journal.pone.0104956 PMC4139310

[nph17436-bib-0016] Dai X , Zhuang Z , Zhao PX . 2018. psRNATarget: a plant small RNA target analysis server (2017 release). Nucleic Acids Research 46: W49–W54. 29718424 10.1093/nar/gky316 PMC6030838

[nph17436-bib-0017] Dean R , Van Kan JAL , Pretorius ZA , Hammond‐Kosack KE , Di Pietro A , Spanu PD , Rudd JJ , Dickman M , Kahmann R , Ellis J *et al*. 2012. The top 10 fungal pathogens in molecular plant pathology. Molecular Plant Pathology 13: 414–430. 22471698 10.1111/j.1364-3703.2011.00783.x PMC6638784

[nph17436-bib-0018] Deng L , Wang H , Sun CL , Li Q , Jiang HL , Du MM , Li CB , Li CY . 2018. Efficient generation of pink‐fruited tomatoes using CRISPR/Cas9 system. Journal of Genetics and Genomics 45: 51–54. 29157799 10.1016/j.jgg.2017.10.002

[nph17436-bib-0019] Dodd AN , Kudla J , Sanders D . 2010. The language of calcium signaling. Annual Review of Plant Biology 61: 593–620. 10.1146/annurev-arplant-070109-104628 20192754

[nph17436-bib-0020] Du M , Zhao J , Tzeng DTW , Liu Y , Deng L , Yang T , Zhai Q , Wu F , Huang Z , Zhou M *et al*. 2017. MYC2 orchestrates a hierarchical transcriptional cascade that regulates jasmonate‐mediated plant immunity in tomato. Plant Cell 29: 1883–1906. 28733419 10.1105/tpc.16.00953 PMC5590496

[nph17436-bib-0021] Dunker F , Trutzenberg A , Rothenpieler JS , Kuhn S , Prols R , Schreiber T , Tissier A , Kemen A , Kemen E , Huckelhoven R *et al*. 2020. Oomycete small RNAs bind to the plant RNA‐induced silencing complex for virulence. eLife 9: e56096. 32441255 10.7554/eLife.56096 PMC7297541

[nph17436-bib-0022] Fang X , Qi Y . 2016. R, e56096NAi in plants: an argonaute‐centered view. Plant Cell 28: 272–285. 26869699 10.1105/tpc.15.00920 PMC4790879

[nph17436-bib-0023] Feng JL , Wang K , Liu X , Chen SN , Chen JS . 2009. The quantification of tomato microRNAs response to viral infection by stem‐loop real‐time RT‐PCR. Gene 437: 14–21. 19374024 10.1016/j.gene.2009.01.017

[nph17436-bib-0024] Feng Q , Li Y , Zhao ZX , Wang WM . 2021. Contribution of small RNA pathway to interactions of rice with pathogens and insect pests. Rice 14: 15. 33547972 10.1186/s12284-021-00458-z PMC7867673

[nph17436-bib-0025] Gao Y , Li SJ , Zhang SW , Feng T , Zhang ZY , Luo SJ , Mao HY , Borkovich KA , Ouyang SQ . 2020. SlymiR482e‐3p mediates tomato wilt disease by modulating ethylene response pathway. Plant Biotechnology Journal 19: 17–19. 32621307 10.1111/pbi.13439 PMC7769227

[nph17436-bib-0026] Goswami RS , Kistler HC . 2004. Heading for disaster*: Fusarium graminearum* on cereal crops. Molecular Plant Pathology 5: 515–525. 20565626 10.1111/j.1364-3703.2004.00252.x

[nph17436-bib-0027] He P , Shan L , Sheen J . 2007. Elicitation and suppression of microbe‐associated molecular pattern‐triggered immunity in plant–microbe interactions. Cellular Microbiology 9: 1385–1396. 17451411 10.1111/j.1462-5822.2007.00944.x

[nph17436-bib-0028] Horbach R , Navarro‐Quesada AR , Knogge W , Deising HB . 2011. When and how to kill a plant cell: Infection strategies of plant pathogenic fungi. Journal of Plant Physiology 168: 51–62. 20674079 10.1016/j.jplph.2010.06.014

[nph17436-bib-0029] Houterman PM , Ma L , van Ooijen G , de Vroomen MJ , Cornelissen BJ , Takken FL , Rep M . 2009. The effector protein Avr2 of the xylem‐colonizing fungus *Fusarium oxysporum* activates the tomato resistance protein I‐2 intracellularly. The Plant Journal 58: 970–978. 19228334 10.1111/j.1365-313X.2009.03838.x

[nph17436-bib-0030] Huang CY , Wang H , Hu P , Hamby R , Jin H . 2019. Small RNAs – big players in plant‐microbe interactions. Cell Host & Microbe 26: 173–182. 31415750 10.1016/j.chom.2019.07.021

[nph17436-bib-0031] Huang J , Ge X , Sun M . 2000. Modified CTAB protocol using a silica matrix for isolation of plant genomic DNA. BioTechniques 28: 434. 10.2144/00283bm08 10723554

[nph17436-bib-0032] Ji H‐M , Zhao M , Gao Y , Cao X‐X , Mao H‐Y , Zhou Y , Fan W‐Y , Borkovich KA , Ouyang S‐Q , Liu P . 2018. FRG3, a target of slmiR482e‐3p, provides resistance against the fungal pathogen *Fusarium oxysporum* in tomato. Frontiers in Plant Science 9: 26. 29434609 10.3389/fpls.2018.00026 PMC5797444

[nph17436-bib-0033] Ji HM , Zhao M , Gao Y , Cao XX , Mao HY , Zhou Y , Fan WY , Borkovich KA , Ouyang SQ , Liu P . 2018b. FRG3, a target of slmiR482e‐3p, provides resistance against the fungal pathogen *Fusarium oxysporum* in tomato. Frontiers in Plant Science 9: 26. 29434609 10.3389/fpls.2018.00026 PMC5797444

[nph17436-bib-0034] Jones JD , Dangl JL . 2006. The plant immune system. Nature 444: 323–329. 17108957 10.1038/nature05286

[nph17436-bib-0035] Katiyar‐Agarwal S , Jin HL . 2010. Role of small RNAs in host–microbe interactions. Annual Review of Phytopathology 48: 225–246. 10.1146/annurev-phyto-073009-114457 PMC3752435 20687832

[nph17436-bib-0036] Kim G , Westwood JH . 2015. Macromolecule exchange in *Cuscuta*‐host plant interactions. Current Opinion in Plant Biology 26: 20–25. 26051214 10.1016/j.pbi.2015.05.012

[nph17436-bib-0037] Kombrink A , Thomma BPHJ . 2013. *LysM* effectors: secreted proteins supporting fungal life. PLoS Pathogens 9: e1003769. 24348247 10.1371/journal.ppat.1003769 PMC3861536

[nph17436-bib-0038] Lakatos L , Szittya G , Silhavy D , Burgyan J . 2004. Molecular mechanism of RNA silencing suppression mediated by p19 protein of tombusviruses. EMBO Journal 23: 876–884. 10.1038/sj.emboj.7600096 PMC381004 14976549

[nph17436-bib-0039] Li B , Gao Y , Mao HY , Borkovich KA , Ouyang SQ . 2019a. The SNARE protein FolVam7 mediates intracellular trafficking to regulate conidiogenesis and pathogenicity in *Fusarium oxysporum* f. sp. lycopersici. Environmental Microbiology 21: 2696–2706. 30848031 10.1111/1462-2920.14585 PMC6850041

[nph17436-bib-0040] Li B , Mao HY , Zhang ZY , Chen XJ , Ouyang SQ . 2019b. FolVps9, a guanine nucleotide exchange factor for FolVps21, is essential for fungal development and pathogenicity in *Fusarium oxysporum* f. sp. *lycopersici* . Frontiers in Microbiology 10: 2658. 31798569 10.3389/fmicb.2019.02658 PMC6868059

[nph17436-bib-0041] Li B , Meng X , Shan L , He P . 2016. Transcriptional regulation of pattern‐triggered immunity in plants. Cell Host & Microbe 19: 641–650. 27173932 10.1016/j.chom.2016.04.011 PMC5049704

[nph17436-bib-0042] Liu P , Duan Y , Liu C , Xue Q , Guo J , Qi T , Kang Z , Guo J . 2018. Corrigendum to: The calcium sensor TaCBL4 and its interacting protein TaCIPK5 are required for wheat resistance to stripe rust fungus. Journal of Experimental Botany 69: 5309. 30165675 10.1093/jxb/ery307

[nph17436-bib-0043] Livak KJ , Schmittgen TD . 2001. Analysis of relative gene expression data using real‐time quantitative PCR and the 2^‐ΔΔCT^ method. Methods 25: 402–408. 11846609 10.1006/meth.2001.1262

[nph17436-bib-0044] Ma X , Nicole MC , Meteignier LV , Hong N , Wang G , Moffett P . 2015. Different roles for RNA silencing and RNA processing components in virus recovery and virus‐induced gene silencing in plants. Journal of Experimental Botany 66: 919–932. 25385769 10.1093/jxb/eru447

[nph17436-bib-0045] Maji RK , Sarkar A , Khatua S , Dasgupta S , Ghosh Z . 2014. PVT: an efficient computational procedure to speed up next‐generation sequence analysis. BMC Bioinformatics 15: 167. 24894600 10.1186/1471-2105-15-167 PMC4063226

[nph17436-bib-0046] Mallory AC , Vaucheret H . 2006. Functions of microRNAs and related small RNAs in plants. Nature Genetics 38: S31–36. 16736022 10.1038/ng1791

[nph17436-bib-0047] McAinsh MR , Pittman JK . 2009. Shaping the calcium signature. New Phytologist 181: 275–294. 10.1111/j.1469-8137.2008.02682.x 19121028

[nph17436-bib-0048] Mendgen K , Hahn M . 2002. Plant infection and the establishment of fungal biotrophy. Trends in Plant Science 7: 352–356. 12167330 10.1016/s1360-1385(02)02297-5

[nph17436-bib-0049] Morel JB , Godon C , Mourrain P , Beclin C , Boutet S , Feuerbach F , Proux F , Vaucheret H . 2002. Fertile hypomorphic *ARGONAUTE* (*ago1*) mutants impaired in post‐transcriptional gene silencing and virus resistance. Plant Cell 14: 629–639. 11910010 10.1105/tpc.010358 PMC150585

[nph17436-bib-0050] Naito Y , Hino K , Bono H , Ui‐Tei K . 2015. CRISPRdirect: software for designing CRISPR/Cas guide RNA with reduced off‐target sites. Bioinformatics 31: 1120–1123. 25414360 10.1093/bioinformatics/btu743 PMC4382898

[nph17436-bib-0051] Navarro L , Dunoyer P , Jay F , Arnold B , Dharmasiri N , Estelle M , Voinnet O , Jones JD . 2006. A plant miRNA contributes to antibacterial resistance by repressing auxin signaling. Science 312: 436–439. 16627744 10.1126/science.1126088

[nph17436-bib-0052] Niu DD , Lii YE , Chellappan P , Lei L , Peralta K , Jiang CH , Guo JH , Coaker G , Jin HL . 2016. miRNA863‐3p sequentially targets negative immune regulator *ARLPK*s and positive regulator *SERRATE* upon bacterial infection. Nature Communications 7: 11324. 10.1038/ncomms11324 PMC4848489 27108563

[nph17436-bib-0053] Ouyang SQ , Park G , Atamian HS , Han CS , Stajich JE , Kaloshian I , Borkovich KA . 2014. MicroRNAs suppress NB domain genes in tomato that confer resistance to *Fusarium oxysporum* . PLoS Pathogens 10: e1004464. 25330340 10.1371/journal.ppat.1004464 PMC4199772

[nph17436-bib-0054] Ouyang SQ , Park G , Ji HM , Borkovich KA . 2021. Small RNA isolation and library construction for expression profiling of small RNAs from *Neurospora crassa* and *Fusarium oxysporum* and analysis of small RNAs in *Fusarium oxysporum*‐infected plant root tissue. Methods in Molecular Biology 2170: 199–212. 32797460 10.1007/978-1-0716-0743-5_14

[nph17436-bib-0055] Pietro AD , Madrid MP , Caracuel Z , Delgado‐Jarana J , Roncero MI . 2003. *Fusarium oxysporum*: exploring the molecular arsenal of a vascular wilt fungus. Molecular Plant Pathology 4: 315–325. 20569392 10.1046/j.1364-3703.2003.00180.x

[nph17436-bib-0056] Qi Y , Denli AM , Hannon GJ . 2005. Biochemical specialization within Arabidopsis RNA silencing pathways. Molecular Cell 19: 421–428. 16061187 10.1016/j.molcel.2005.06.014

[nph17436-bib-0057] Qi YJ , He XY , Wang XJ , Kohany O , Jurka J , Hannon GJ . 2006. Distinct catalytic and non‐catalytic roles of ARGONAUTE4 in RNA‐directed DNA methylation. Nature 443: 1008–1012. 16998468 10.1038/nature05198

[nph17436-bib-0058] Qi Y , Mi S . 2010. Purification of Arabidopsis argonaute complexes and associated small RNAs. Methods in Molecular Biology 592: 243–254. 19802600 10.1007/978-1-60327-005-2_16

[nph17436-bib-0059] Ruiz‐Ferrer V , Voinnet O . 2009. Roles of plant small RNAs in biotic stress responses. Annual Review of Plant Biology 60: 485–510. 10.1146/annurev.arplant.043008.092111 19519217

[nph17436-bib-0060] Schwessinger B , Ronald PC . 2012. Plant innate immunity: perception of conserved microbial signatures. Annual Review of Plant Biology 63: 451–482. 10.1146/annurev-arplant-042811-105518 22404464

[nph17436-bib-0061] Shahid S , Kim G , Johnson NR , Wafula E , Wang F , Coruh C , Bernal‐Galeano V , Phifer T , dePamphilis CW , Westwood JH *et al*. 2018. MicroRNAs from the parasitic plant *Cuscuta campestris* target host messenger RNAs. Nature 553: 82–85. 29300014 10.1038/nature25027

[nph17436-bib-0062] Simons G , Groenendijk J , Wijbrandi J , Reijans M , Groenen J , Diergaarde P , Van der Lee T , Bleeker M , Onstenk J , de Both M *et al*. 1998. Dissection of the Fusarium *I2* gene cluster in tomato reveals six homologs and one active gene copy. Plant Cell 10: 1055–1068. 9634592 10.1105/tpc.10.6.1055 PMC144031

[nph17436-bib-0063] Singh J , Mishra V , Wang F , Huang HY , Pikaard CS . 2019. Reaction mechanisms of Pol IV, RDR2, and DCL3 drive RNA channeling in the siRNA‐Directed DNA methylation pathway. Molecular Cell 75: 576–589. 31398324 10.1016/j.molcel.2019.07.008 PMC6698059

[nph17436-bib-0064] Steinhorst L , Kudla J . 2013. Calcium and reactive oxygen species rule the waves of signaling. Plant Physiology 163: 471–485. 23898042 10.1104/pp.113.222950 PMC3793029

[nph17436-bib-0065] Stergiopoulos I , de Wit PJGM . 2009. Fungal effector proteins. Annual Review of Phytopathology 47: 233–263. 10.1146/annurev.phyto.112408.132637 19400631

[nph17436-bib-0066] Tang RJ , Wang C , Li K , Luan S . 2020. The CBL‐CIPK calcium signaling network: unified paradigm from 20 years of discoveries. Trends in Plant Science 25: 604–617. 32407699 10.1016/j.tplants.2020.01.009

[nph17436-bib-0067] Validov SZ , Kamilova FD , Lugtenberg BJ . 2011a. Monitoring of pathogenic and non‐pathogenic *Fusarium oxysporum* strains during tomato plant infection. Microbial Biotechnology 4: 82–88. 21255375 10.1111/j.1751-7915.2010.00214.x PMC3815798

[nph17436-bib-0068] Validov SZ , Kamilova FD , Lugtenberg BJJ . 2011b. Monitoring of pathogenic and non‐pathogenic *Fusarium oxysporum* strains during tomato plant infection. Microbial Biotechnology 4: 82–88. 21255375 10.1111/j.1751-7915.2010.00214.x PMC3815798

[nph17436-bib-0069] Varkonyi‐Gasic E , Wu RM , Wood M , Walton EF , Hellens RP . 2007. Protocol: a highly sensitive RT‐PCR method for detection and quantification of microRNAs. Plant Methods 3: 12. 17931426 10.1186/1746-4811-3-12 PMC2225395

[nph17436-bib-0070] Vaucheret H . 2006. Post‐transcriptional small RNA pathways in plants: mechanisms and regulations. Genes & Development 20: 759–771. 16600909 10.1101/gad.1410506

[nph17436-bib-0071] Vogel HJ . 1956. A convenient growth medium for *Neurospora crassa* . Microbial Genetics Bulletin 13: 42–47.

[nph17436-bib-0072] Wang B , Sun Y , Song N , Zhao M , Liu R , Feng H , Wang X , Kang Z . 2017. *Puccinia striiformis* f. sp. *tritici* microRNA‐like RNA 1 (*Pst*‐milR1), an important pathogenicity factor of *Pst*, impairs wheat resistance to *Pst* by suppressing the wheat pathogenesis‐related 2 gene. New Phytologist 215: 338–350. 10.1111/nph.14577 28464281

[nph17436-bib-0074] Wang M , Weiberg A , Dellota E Jr , Yamane D , Jin H . 2017. Botrytis small RNA *Bc*‐siR37 suppresses plant defense genes by cross‐kingdom RNAi. RNA Biology 14: 421–428. 28267415 10.1080/15476286.2017.1291112 PMC5411126

[nph17436-bib-0073] Wang M , Weiberg A , Lin FM , Thomma BP , Huang HD , Jin H . 2016. Bidirectional cross‐kingdom RNAi and fungal uptake of external RNAs confer plant protection. Nature Plants 2: 16151. 27643635 10.1038/nplants.2016.151 PMC5040644

[nph17436-bib-0075] Weiberg A , Wang M , Lin FM , Zhao H , Zhang Z , Kaloshian I , Huang HD , Jin H . 2013. Fungal small RNAs suppress plant immunity by hijacking host RNA interference pathways. Science 342: 118–123. 24092744 10.1126/science.1239705 PMC4096153

[nph17436-bib-0076] Wu L , Zhang Q , Zhou H , Ni F , Wu X , Qi Y . 2009. Rice microRNA effector complexes and targets. Plant Cell 21: 3421–3435. 19903869 10.1105/tpc.109.070938 PMC2798332

[nph17436-bib-0077] Zhang T , Zhao YL , Zhao JH , Wang S , Jin Y , Chen ZQ , Fang YY , Hua CL , Ding SW , Guo HS . 2016. Cotton plants export microRNAs to inhibit virulence gene expression in a fungal pathogen. Nature Plants 2: 16153. 27668926 10.1038/nplants.2016.153

[nph17436-bib-0078] Zilberman D , Cao XF , Jacobsen SE . 2003. *ARGONAUTE4* control of locus‐specific siRNA accumulation and DNA and histone methylation. Science 299: 716–719. 12522258 10.1126/science.1079695

