# Supplementary material for: Fol‐milR1, a pathogenicity factor of Fusarium oxysporum, confers tomato wilt disease resistance by impairing host immune responses
Source: New Phytol. 2021 May 30;232(2):705–18. doi: 10.1111/nph.17436 (PMC8518127; doi:10.1111/nph.17436)
Supplement: Supplementary file 1 — Fig. S1 The grades of wilt disease severity. Fig. S2 The abundance of Fol increases with time in both tomato cultivars, but to a much greater extent in ‘Moneymaker’. Fig. S3 Targeted gene replacement of Fol‐milR1 in Fol. Fig. S4 Fol‐milR1 expression levels in knockout and overexpression transformants, as measured using stem‐loop quantitative real‐time polymerase chain reaction (qRT‐PCR). Fig. S5 Growth and colonial morphology of the Fol‐milR1‐KO and Fol‐milR1‐OE strains in response to various stressors. Fig. S6 The 5′RLM‐RACE polymerase chain reaction products used for sequencing. Fig. S7 Wilt disease symptoms of VIGS‐SlyAGOs and control plants infected by Fol. Fig. S8 Comparison of homologous regions in SlyAGO4a and AtAGO4. Fig. S9 No association can be detected between Fol‐milR1 and SlyAGO1. [file NPH-232-705-s006.pdf]

“New Phytologist Supporting Information”, *Fol*-milR1, a pathogenicity factor of *Fusarium oxysporum*, confers tomato wilt disease resistance by impairing host immune responses. Hui-Min Ji, Hui-Ying Mao, Si-Jian Li, Tao Feng, Zhao-Yang Zhang, Lu Cheng, Shu-Jie Luo, Katherine A. Borkovich and Shou-Qiang Ouyang. 14 April, 2021.

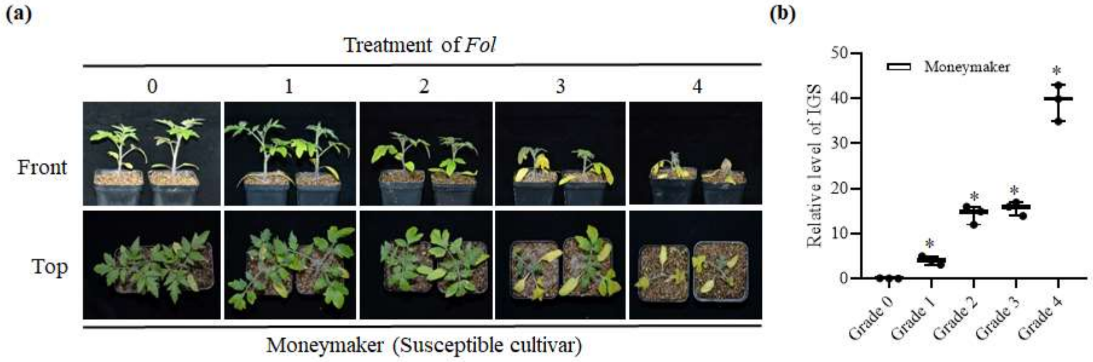

Supplementary Fig. 1 The grades of wilt disease severity. Two-week-old susceptible cultivar Moneymaker tomato seedlings were removed from soil and roots incubated for 30 min in a solution of *Fol* conidia at a concentration of $1X10^8$ /ml. Water-treated tomato seedlings were used as the grade 0. The grades were scaled at 3 weeks after inoculation.

(a) Wilt disease symptoms of Moneymaker plants.

(b) Relative levels of fungal ribosomal intergenic spacer region (IGS) amplified from genomic DNA correlate with *Fol* biomass in Moneymaker plants. * indicates significant difference when compared to the corresponding control plants in the same treatments at $P < 0.05$, chi-square test; Error bars indicate the Standard Deviation of three replicates.

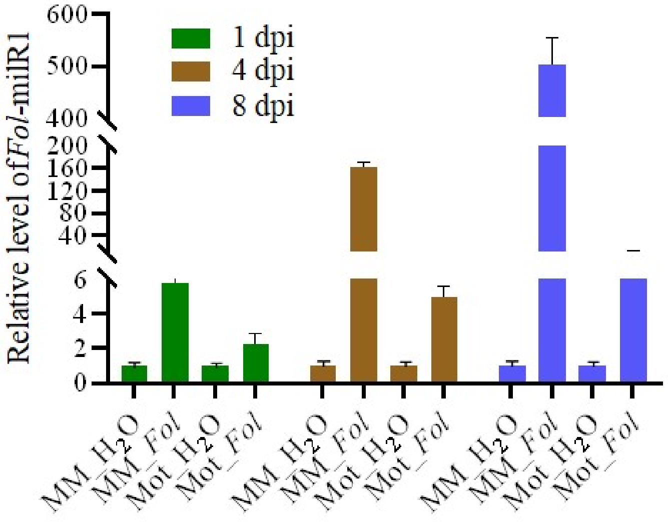

Supplementary Fig. 2 The abundance of *Fol* increases with time in both tomato cultivars, but to a much greater extent in Moneymaker. Two-week-old tomato seedlings were removed from soil and roots incubated for 30 min in a solution of *Fol*

conidia at a concentration of 1 × 10^8 /ml. Water-treated tomato seedlings were used as the negative control. Roots were collected at 1 dpi, 4 dpi and 8 dpi, respectively. In order to control for experimental variation, all experiments were repeated three times. Error bars indicate the Standard Deviation of three replicates.

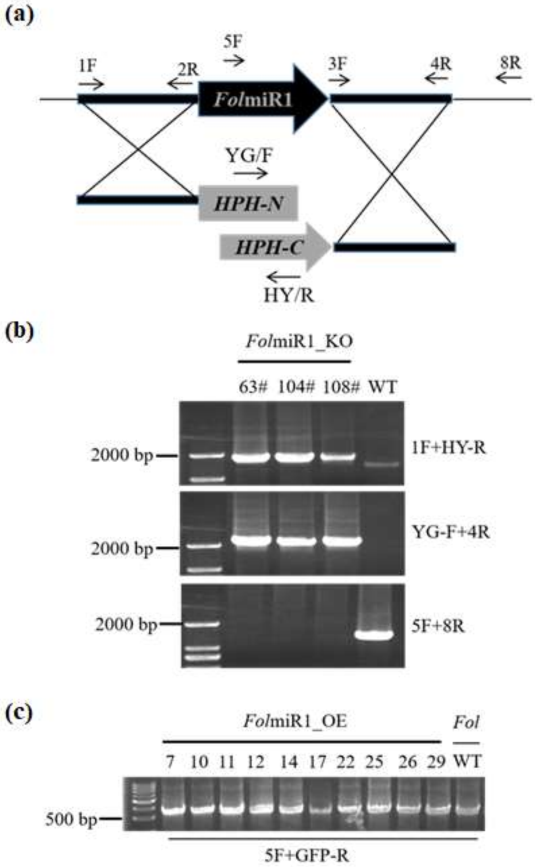

Supplementary Fig. 3 Targeted gene replacement of *Fol*-milR1 in *Fol*.

(a) Schematic diagram of the split-marker gene deletion strategy for *Fol*-milR1.

(b) Identification of *Fol*-milR1-KO transformants using specific primers in PCRs.

(c) Identification of *Fol*-milR1-OE transformants by specific primers using PCR.

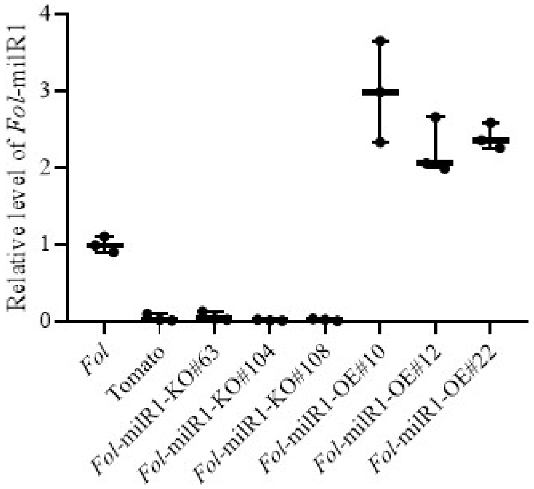

Supplementary Fig. 4 The expression level of *Fol*-milR1 in knockout and overexpression transformants by stem-loop qRT-PCR. Error bars indicate the Standard Deviation of three replicates.

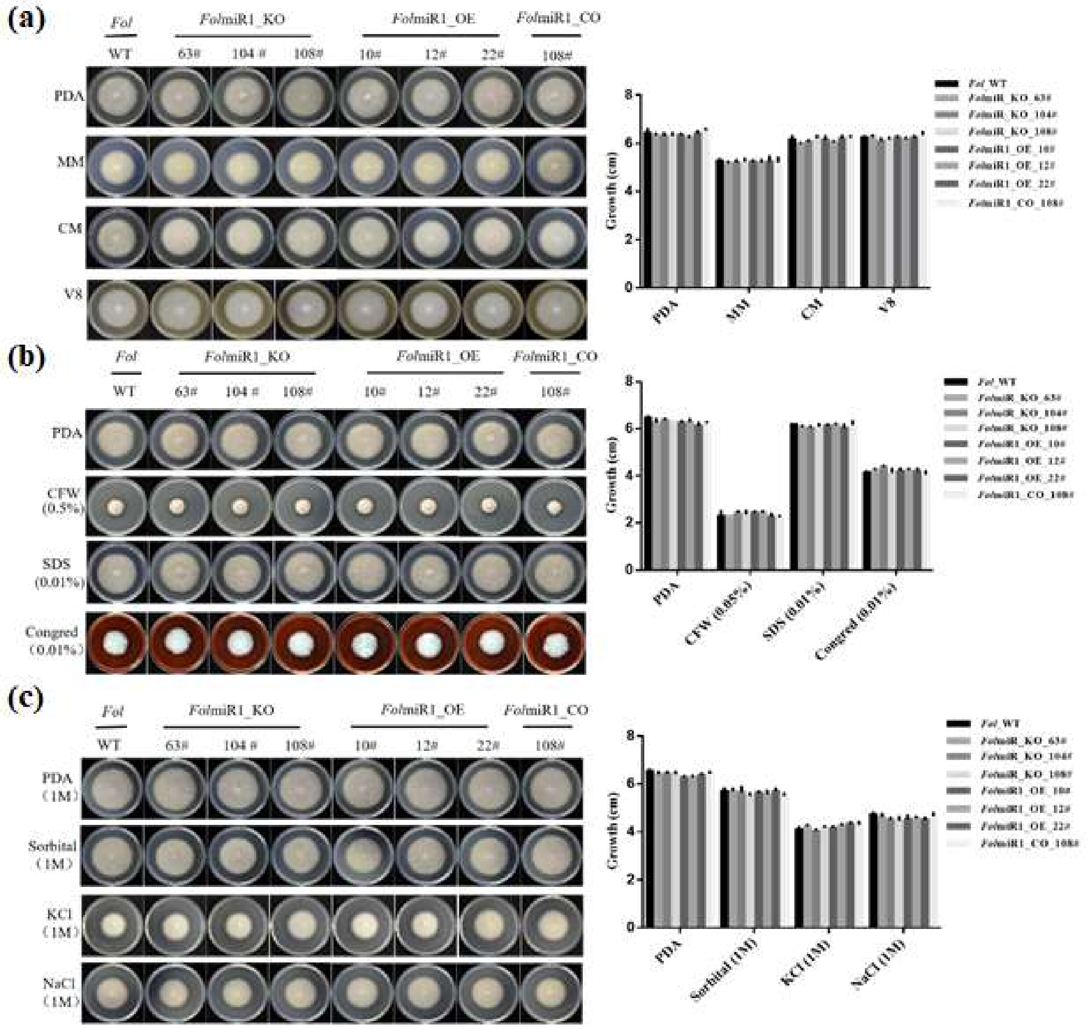

Supplementary Fig. 5 Growth and colonial morphology of the *Fol*-milR1-KO and *Fol*-milR1-OE strains in response to various stressors.

(a) The indicated *Fol* strains were inoculated on PDA, MM, CM and V8 medium plates at 28 ℃, and photographed after 7 days. WT, wild type. KO, Knockout strain. OE, Overexpression strain. CO, complemented strain.

(b) *Fol* strains were inoculated on PDA medium plates supplemented with the cell wall antagonists Calcofluor white (CFW; 0.05%), Congo red CR (CR; 0.05%) or Sodium dodecyl sulfate (SDS; 0.01%) at 28 ℃, and photographed after 7 days.

(c) Fol strains were inoculated on PDA medium plates supplemented with hyperosmotic stressors sorbitol (1 M), sodium chloride (NaCl, 1 M) or potassium chloride (KCl, 1 M) at 28 ℃, and photographed after 7 days.

* indicates significant difference when compared to the corresponding control plants in

the same treatments at P < 0.05, chi-square test; Error bars indicate the Standard Deviation of three replicates.

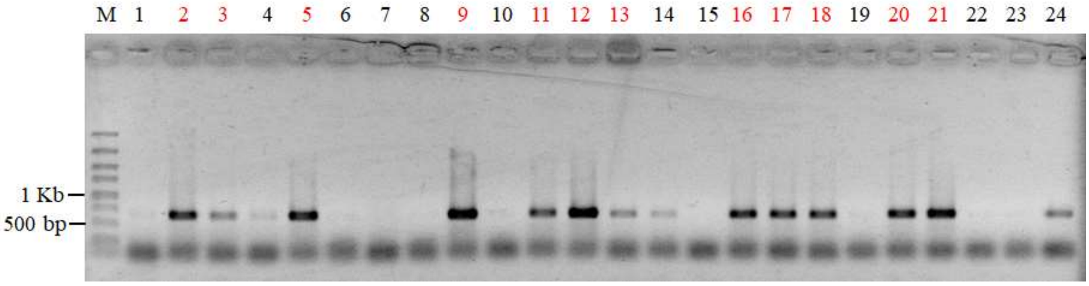

Supplementary Fig. 6 The 5' RLM-RACE PCR products used for sequencing. Total RNA was isolated from the *N. benthamiana* leaves used for co-expression of *Fol*-milR1 and the *SlyFRG4* target. mRNAs were prepared using two rounds of purification with an Oligotex mRNA Midi Kit (#70042 Qiagen) and directly ligated to the FirstChoice RLMRACE Kit RNA Oligo adaptor without further modifications. Gene-specific primers were designed approximately 600 nucleotides to the 3' side of predicted target sites. The conditions used for this amplification step were those for gene specific RACE as recommended by the manufacturer. A total of 24 clones were verified by diagnostic PCR and the 12 clones labeled using red font were sequenced.

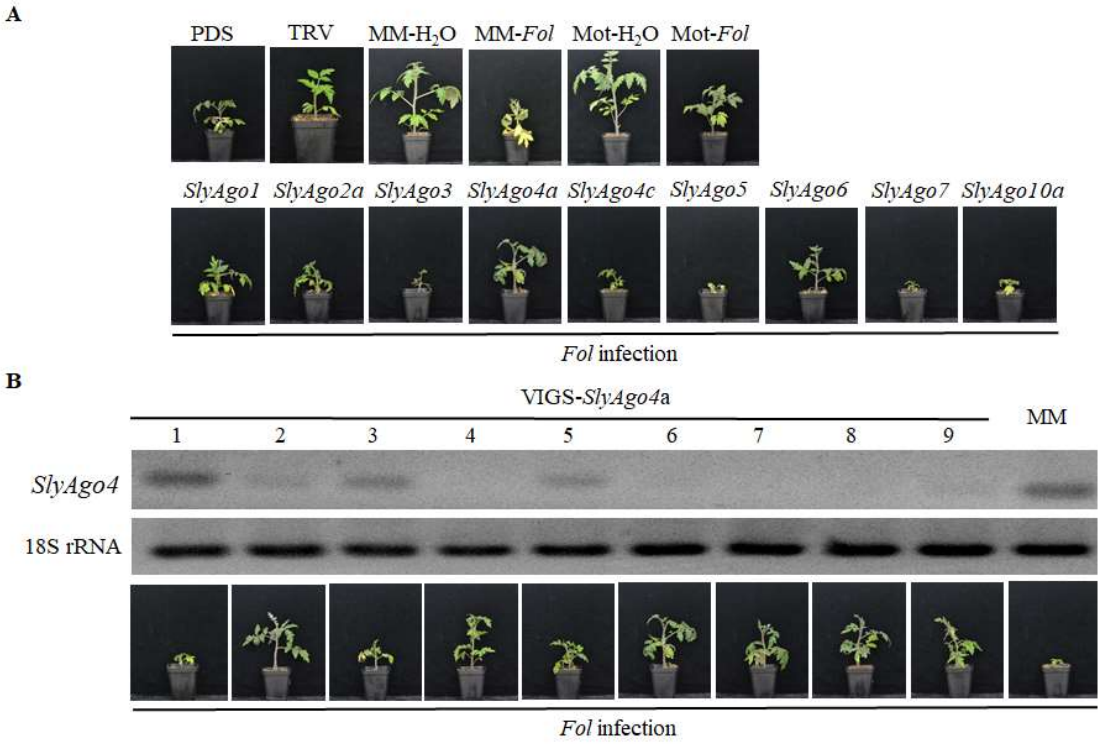

Supplementary Fig. 7 Wilt disease symptoms of VIGS-*SlyAGOs* and control plants infected by *Fol*.

(a) VIGS was used to down-regulate expression of the 9 indicated *SlyAGO*s in tomato. VIGS-*SlyAGO*s plants were treated with water or *Fol* and photographed 2 weeks later. *Phytoene Desaturase* (*PDS*) TRV-silenced plant (TRV-*PDS*) and TRV-vector plant were used as positive control for silencing.

(b) The expression levels of *SlyAGO4* were checked by RT-PCR in selected VIGS-*SlyAGO4* plants. VIGS-*SlyAGO4* plants were treated with water or *Fol* and photographed 2 weeks later.

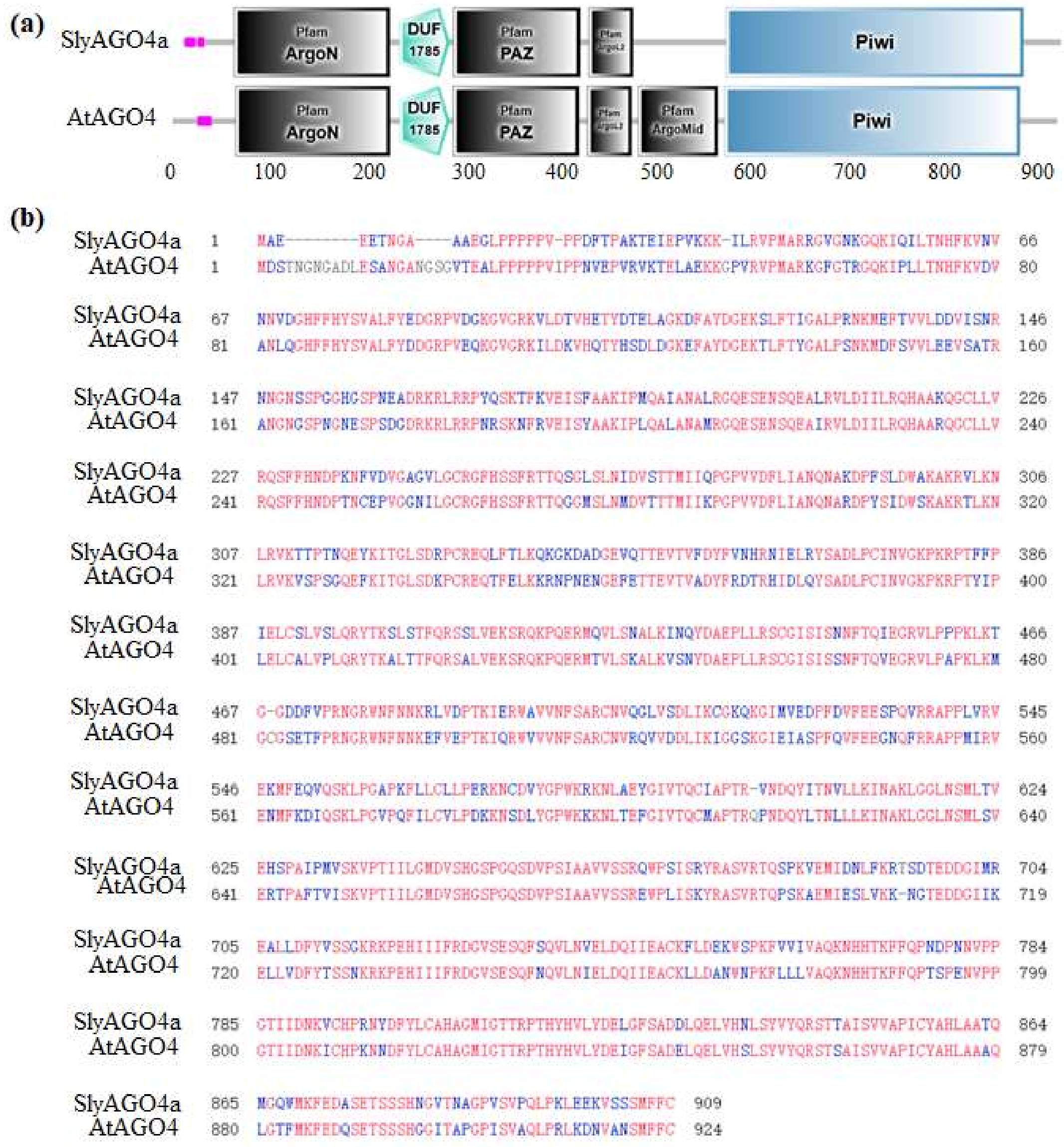

Supplementary Fig. 8 Comparison of homologous regions in SlyAGO4a and AtAGO4.

(a) Predicted protein domains of SlyAGO4a (SOLYC01G008960) and AtAGO4 (AT2G27040).

(b) Alignment of the amino acid sequences of SlyAGO4a and AtAGO4.

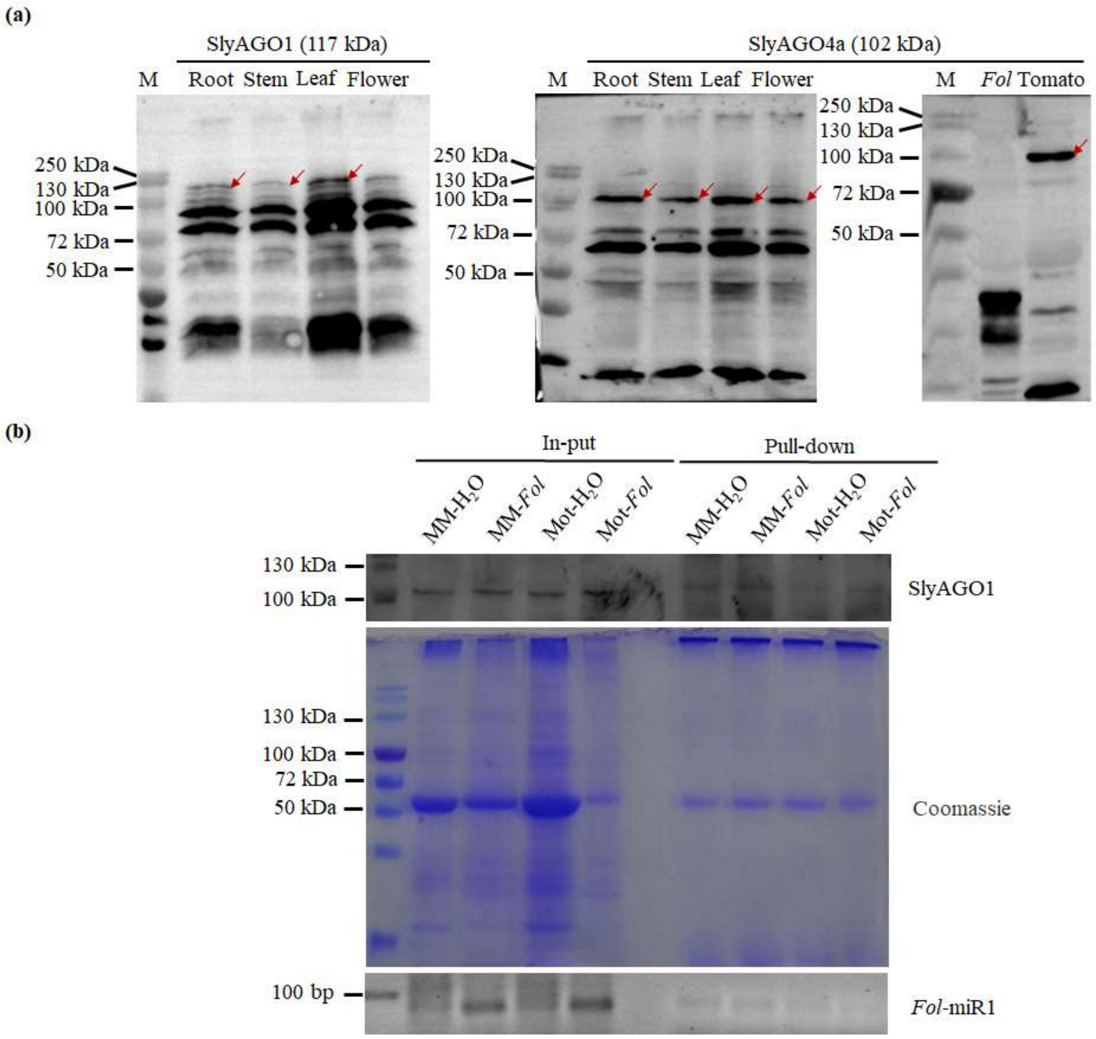

Supplementary Fig. 9 No association can be detected between *Fol*-milR1 and SlyAGO1.

(a) SlyAGO1 and SlyAGO4a antibodies react with proteins of the predicted sizes (indicated by red arrows) during western analysis of protein extracts from tomato.

(b) No association can be detected between *Fol*-milR1 and SlyAGO1 after immunoprecipitation of tomato extracts with SlyAGO1 antibody and RT-PCR using *Fol*-milR1 primers.
